# The Efficacy and Safety of Chinese Herbal Medicine Jinlida as Add-On Medication in Type 2 Diabetes Patients Ineffectively Managed by Metformin Monotherapy: A Double-Blind, Randomized, Placebo-Controlled, Multicenter Trial

**DOI:** 10.1371/journal.pone.0130550

**Published:** 2015-06-22

**Authors:** Fengmei Lian, Jiaxing Tian, Xinyan Chen, Zhibin Li, Chunli Piao, Junjie Guo, Licheng Ma, Lijuan Zhao, Chengdong Xia, Chong-Zhi Wang, Chun-Su Yuan, Xiaolin Tong

**Affiliations:** 1 Guang’anmen Hospital, China Academy of Chinese Medical Sciences, Beijing, China; 2 Shijiazhuang Hospital of Traditional Chinese Medicine, Shijiazhuang, China; 3 The Affiliated Hospital to Changchun University of Chinese Medicine, Changchun, China; 4 Shanxi Province Hospital of Traditional Chinese Medicine, Taiyuan, China; 5 Bethune International Peace Hospital, Shijiazhuang, China; 6 The Affiliated Hospital to Shanxi University of Traditional Chinese Medicine, Taiyuan, China; 7 Xiyuan Hospital, China Academy of Chinese Medical Sciences, Beijing, China; 8 Tang Center for Herbal Medicine Research, University of Chicago, Chicago, IL, United States of America; Weill Cornell Medical College Qatar, QATAR

## Abstract

**Background:**

Metformin plays an important role in diabetes treatment. Studies have shown that the combined use of oral hypoglycemic medications is more effective than metformin monotherapy. In this double-blind, randomized, placebo-controlled, multicenter trial, we evaluated whether Jinlida, a Chinese herbal medicine, enhances the glycemic control of metformin in type 2 diabetes patients whose HbA1c was ineffectively controlled with metformin alone.

**Methods:**

A total of 186 diabetes patients were enrolled in this double-Blind, randomized, placebo-controlled, multicenter trial. Subjects were randomly allocated to receive either Jinlida (9 g) or the placebo TID for 12 consecutive weeks. All subjects in both groups also continuously received their metformin without any dose change. During this 12-week period, the HbA1c, FPG, 2h PG, body weight, BMI were assessed. HOMA insulin resistance (HOMA-IR) and β-cell function (HOMA- β) were also evaluated.

**Results:**

At week 12, compared to the HbA1c level from week 0, the level of the Jinlida group was reduced by 0.92 ± 1.09% and that of the placebo group was reduced by 0.53 ± 0.94%. The 95% CI was 0.69 - 1.14 for the Jinlida group vs. 0.34 - 0.72 for the placebo group. There was a very significant HbA1c reduction between the two groups after 12 weeks (p < 0.01). Both FG and 2h PG levels of the Jinlida group and placebo group were reduced from week 0. There were a very significant FG and 2h PG level reductions between the two groups after 12 weeks (both p < 0.01). The Jinlida group also showed improved β-cell function with a HOMA-β increase (p < 0.05). No statistical significance was observed in the body weight and BMI changes. No serious adverse events were reported.

**Conclusion:**

Jinlida significantly enhanced the hypoglycemic action of metformin when the drug was used alone. This Chinese herbal medicine may have a clinical value as an add-on medication to metformin monotherapy.

**Trial Registration:**

Chinese Clinical Trial Register ChiCTR-TRC-13003159

## Introduction

Diabetes is a chronic metabolic disease that seriously affects patients worldwide. It is estimated that there will be over 366 million people suffering from this disease by 2030 [1] With fast economic development and lifestyle changes, China has the highest number of diabetic patients in the world. Currently there are 92.4 million diabetic adults in China, and another 1.5 million adults are in the pre-diabetic stage [2]. Several large-scale clinical trials, such as the United Kingdom Prospective Diabetes Study (UKPDS), have shown that good glycemic control is critical for patients with type 2 diabetes, since the HbA1c levels are correlated with diabetic complications [3,4]. The American Diabetes Association and the Chinese Diabetes Society adopt HbA1c < 7.0% as the goal to control for individuals with type 2 diabetes [5,6]. However, studies have shown that, among Chinese patients who take oral diabetic medications, more than two thirds could not effectively control their HbA1c levels [7].

Metformin plays an important role in type 2 diabetes treatment worldwide, including those patients in China. If metformin alone cannot effectively control the HbA1c to a desirable level, other oral antidiabetic drugs should be added as a combination therapy [5,6]. Previous large-scale clinical studies have demonstrated that the combined use of oral hypoglycemic medications is more effective than antidiabetic monotherapy [8–10].

Traditional Chinese medicine (TCM) has a potential in the prevention and treatment of type 2 diabetes [11]. Our group has reported the efficacy and safety of several Chinese herbal medicines that effectively reduced blood glucose and HbA1c levels in diabetic and pre-diabetic patients [12–14]. Thus, we believe that Chinese herbal medicine may play a role in treating this very common metabolic disease.

Jinlida is a Chinese herbal drug used in China for diabetes management with positive data reported in Chinese literature from animal research and human studies [15–18]. However, the published antidiabetes clinical trials of Jinlida were not large-scale multi-center controlled trials. Treatment of diabetes often requires the use of a combination of different medications. For those patients whose glycemic level could not be effectively controlled with metformin alone, Jinlida could be used as an add-on therapy. In this double-blind, randomized, placebo-controlled, multicenter trial, we evaluated whether Jinlida enhances glycemic control of metforminin in type 2 diabetes patients whose diabetes was poorly managed with metformin monotherapy.

## Materials and Methods

This study used a randomized, double-blind, placebo-controlled, multicenter design. Data were collected across seven clinical research centers (sites) in China. The research protocol was approved by the Guang’anmen Hospital Medical Ethics Commission in China. Written, informed consent was obtained from each study subject. The clinical trial registration number of this study is ChiCTR-TRC-13003159 (www.chictr.org/cn/).

### Subjects

Patients that met the following criteria were eligible for this study: (1) were 18–70 years old, (2) were diagnosed as type 2 diabetics based WHO type 2 diabetes diagnostic criteria [19]; (3) had undergone standard diet control, exercise therapy; (4) had been receiving metformin in a steady dose for over three months; (5) had HbA1c ≥ 7.0%; (6) had fasting plasma glucose (FPG) 7.0–13.9 mmol/L, or 2 h postprandial glucose (2h PG) ≥ 11.1 mmol/L; (7) had a body mass index (BMI) >18 but < 40 kg/m^2^.

Patients were excluded from the study if they met one of the following conditions: (1) had type 1 diabetes, gestational diabetes, or other specific types of diabetes; (2) had used a weight control medication in the past three months or had taken any oral antidiabetic drug or insulin in addition to metformin; (3) had serious gastrointestinal tract diseases, such as gastric ulcers, GI tract bleeding, gastroparesis, pyloric stenosis, or had received gastric bypass surgery; (4) experienced stressful situations such as diabetic ketoacidosis, nonketotic hyperosmolar diabetic coma, severe infection, or surgery in the previous month; (5) had abnormal ECG and routine blood test; (6) suffered from severe hepatic insufficiency or renal insufficiency with serum creatinine (Cr) > 132.6 μmol/L (1.5 mg/dL); (7) had uncontrolled hypertension (blood pressure or BP ≥ 160/100 mmHg); (8) had high blood lipids or triglycerides (TG) > 5.65 mmol/L; (9) had complications of diabetes; (10) had mental illness, abused or addicted to alcohol, psychoactive substances or other drugs; (11) was pregnant, lactating, or planned to become pregnant; (12) had a history of medication allergies.

**Fig 1:**
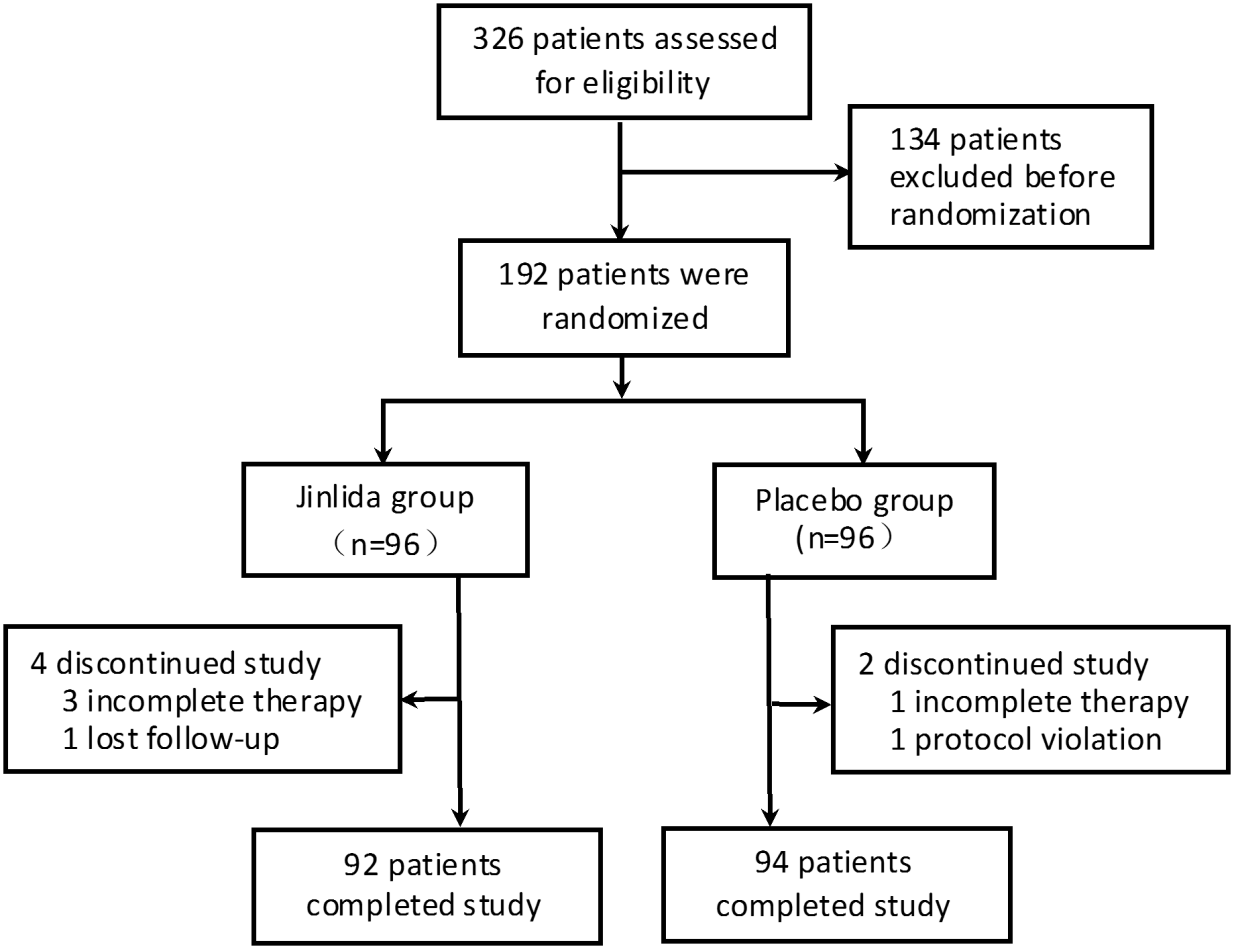
Flow diagram of participant screening, randomization, and treatment. Four subjects in the Jinlida group and 2 subjects in the placebo group were excluded from the study due to various reasons listed.

Based on our previous clinical practice using Jinlida in the treatment in this study patient population, i.e., type 2 diabetes patients ineffectively controlled with metformin monotherapy, we noticed that the HbA1c level generally reduced 0.5% or more, and this observation was supported by a pilot study in five patients. Thus, with HbA1c 0.5% reduction with Jinlida, we need to enroll 77 subjects per group to ensure an 80% power to detect that the Jinlida group has a significantly better effect on HbA1c than that of placebo group. Considering factors such as dropouts, 96 subjects were recruited per group (Fig 1, S1 File).

Between April 2013 and October 2013, a total of 192 subjects were enrolled from seven clinical research sites after screening.

Patients who met the following criteria were asked to withdraw from the trial: (1) experienced serious diabetic complications (such as diabetic ketoacidosis, hyperosmolar nonketotic syndrome, lactic acidosis, hypoglycemic coma); (2) after 4 weeks of the start of the trial, FPG level was > 13.9 mmol/L in two consecutive tests within a week; (3) had poor compliance, with test medication use less than 80% of or more than 120% of the prescribed dose; (4) had violated protocol, such as taking another oral hypoglycemic agent.

### Study medication

The Jinlida, in one batch number, manufactured by Shijiazhuang Yiling Pharmaceutical Co. (Shijiazhuang, China), was used. This herbal drug, prepared in small granules, is brown to dark brown in color and has a bitter taste. The Jinlida consists of 17 Chinese medicinal herbs, including ginseng, polygonati, atractylodis lanceae, sophorae flavescentis, ophiopogon japonicus, rehmanniae, polygoni multiflori, dogwood, poria, perrin, coptis chinensis, anemarrhena, epimedium, salvia, puerariae, semen litchi, and cortex lycii radicis. The quality of these herbs and decoction preparation was in accordance with the Chinese Pharmacopoeia (2005). The placebo was composed of malt, dextrin and lactose, also prepared as granules. The color, odor, shape, taste and packaging of the placebo were basically identical to that of Jinlida. The Jinlida and placebo were supplied by the manufacturer free of charge, but the manufacturer was not involved in the design and analysis of this study.

### Chemical analysis of Jinlida

The chemical composition of Jinlida was analyzed using an ultra-performance liquid chromatography/mass (UPLC/MS) method. The UPLC system was a Waters ACQUITY instrument (Milford, MA), with a Waters Synapt High Definition MS System, and MassLynx V4.1software for peak identification and integration. The separation was carried out on a Waters HSS T3 column (1.8 μ, 100 × 2.1 mm I.D.). For UPLC analysis, a 5-μl test sample was injected into the column and eluted at 25°C with a constant flow rate of 0.3 ml/min. In gradient elution the composition of the mobile phases are (solvent A): water with 0.1% formic acid, and (solvent B): acetonitrile with 0.1% formic acid. Chemical standards and Jinlida capsule powder were dissolved in methanol. All solutions were filtered through Millex 0.22 μm nylon membrane syringe filters before use.

**Fig 2:**
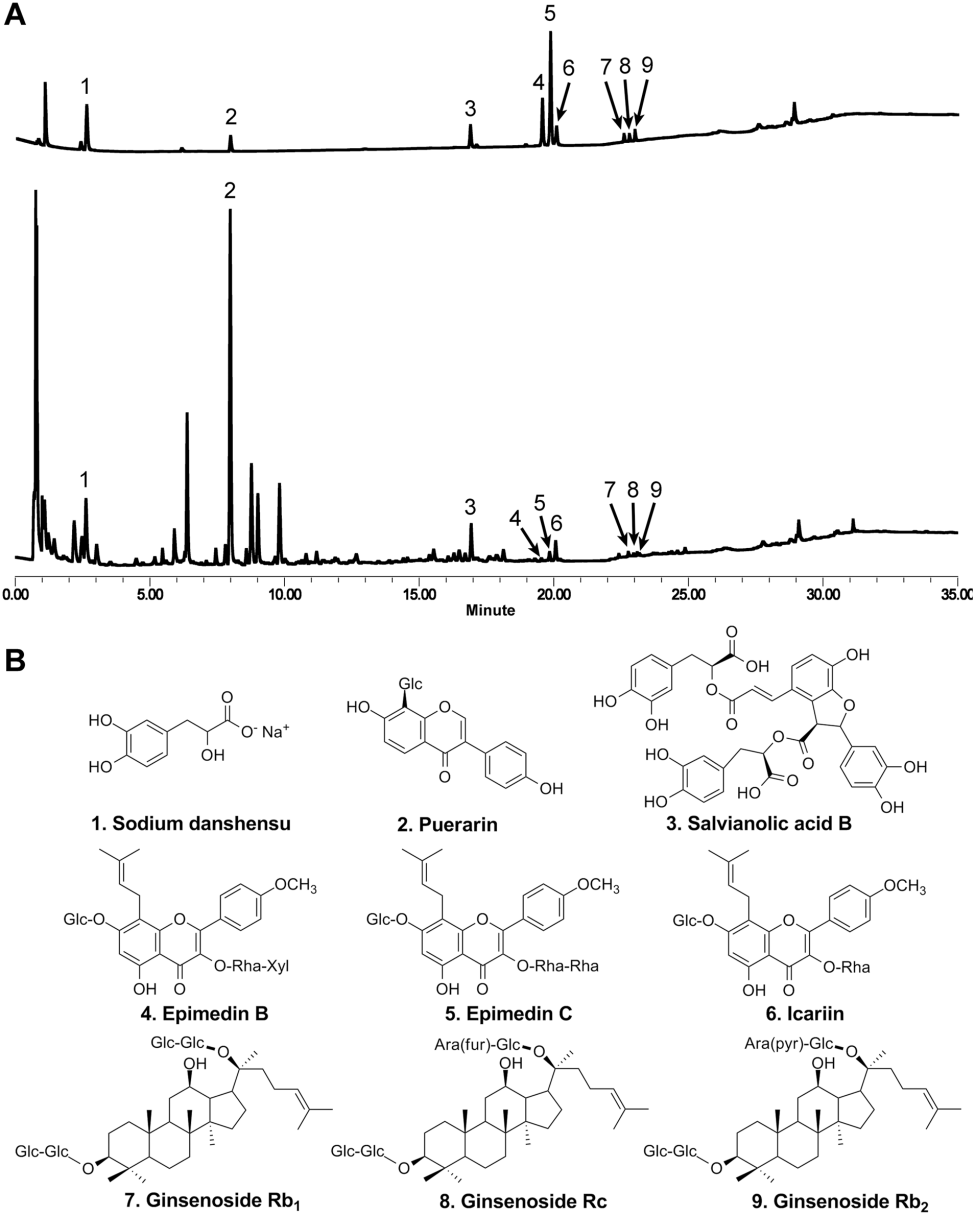
Ultra-performance liquid chromatography/mass spectrometry (UPLC/MS) analysis of Jinlida. (A) Representative total ion current chromatograms of 9 standards (upper panel) and Jinlida (lower panel). (B) Chemical structures of identified compounds in Jinlida, corresponding to the peak numbers indicated in the chromatograms.

The nine chemical standards, including sodium dashensu, puerarin, salvianolic acid B, epimedin B, epimedin C, icariin, ginsenoside Rb1, ginsenoside Rc, and ginsenoside Rb2, were used as the quality control markers in Jinlida. Representative chromatograms of standards and tested drug and chemical structures of marker compounds are shown in Fig 2.

### Intervention and efficacy evaluation

The subjects were randomly allocated to receive either Jinlida or the placebo for 12 consecutive weeks. Subjects in both the Jinlida and placebo groups were orally administered one bag of granules (9 g) three times daily with warm water before each meal. All subjects in both groups also continuously received their metformin without any dose change. The average daily metformin dose of the subjects in the Jinlida group and placebo group was 1,210 mg and 1,310 mg, respectively.

During this 12-week period, subjects were assessed at 0, 4, 8 and 12 weeks. In each session, subjects were asked if there were any adverse events. All subjects received a symptom assessment, physical examination, and the compliance of the test drug administration. The HbA1c was measured at 0 and 12 weeks. The FPG was measured at 0, 4, 8 and 12 weeks. 2h PG was measured at 0, 4 and 12 weeks. Body weight, BMI and waist size were also monitored.

The primary endpoints were the changes in HbA1c, FPG and 2h PG levels of the two groups after 12 weeks for this Jinlida add-on trial, in the type 2 diabetic subjects ineffectively controlled with metformin monotherapy. The HbA1c level was measured in a central laboratory (Guang’anmen Hospital, China Academy of Chinese Medical Sciences) using an ADAMSPMALCHA-8160 automated HbA1c analyzer (Japan). The secondary endpoints were changes in the insulin resistance, β-cell function, body weight and BMI between the two groups. At 0, 4 and 12 weeks, plasma insulin levels were measured, and the homeostatic model assessment (HOMA) was performed to quantify insulin resistance (HOMA-IR) and β-cell function (HOMA-β).

### Randomization and blinding

A stratified, block randomization method was conducted by the study center. Study drugs were packed and numbered according to the random coding form and randomly allocated to each research site using concealed opaque envelopes. These envelopes and case report forms were not collected until the end of the trial. Study drugs were provided based on the assigned numbers, which were determined according to the visit sequence and study drug number sequence, and remained unchanged throughout the trial. During the trial, neither the clinicians nor the patients were aware of the grouping. The only basis of drug distribution was the unique drug number. Independent statisticians performed the data analysis.

### Safety monitoring

Based on previous reported clinical trials and observational human experience, the test herbal medication, Jinlida, was well tolerated and not associated with any safety issues. We thus considered the overall level of risk of the clinical study to be low. During the study, all adverse experiences were monitored and recorded on the case report form with special notes made on the time of onset and resolution, severity, and the investigator’s analysis of the relationship between the adverse experience and the test drug. The safety procedures were also in place when the proposal was approved by the local Medical Ethics Commission in China. A Data and Safety Monitoring Board was formed, including physicians experienced in this research area and a biostatistician. The board was responsible for oversight all issues related to the safety of the study subjects.

### Statistical analysis

Data entry was completed twice by two staff members using SPSS19.0 software (SPSS Inc., Chicago, IL). Data were summarized as means ± S.D. or otherwise indicated. Categorical data were presented as frequencies. For between-group comparison, *t*-test or Wilcoxon rank sum test was performed. When examining the central effects and covariates, the analysis of covariance was used. χ^2^ test or Fisher's exact test were used to compare the incidence of adverse events between the two groups. The level of statistical significance was set at p < 0.05.

## Results

### Subject characteristics

There were 326 subjects who participated in the initial screening and 192 eligible subjects entered the randomization. 186 subjects completed the study including 92 in the Jinlida group and 94 in the placebo group. The remaining 6 subjects (4 in the Jinlida group and 2 in placebo group) were excluded from the study due to incomplete treatment, lost of follow-up, or protocol violation (Fig 1, S2 File and S1 CONSORT Checklist).

The baseline characteristics of the subjects are shown in Table 1. The mean HbA1c (%) in both the Jinlida and placebo groups was 8.10 ± 0.89 and 8.33 ± 1.22, respectively (p = 0.15). The stable daily metformin dose (g) in both the Jinlida and placebo groups was 1.21 ± 0.47 and 1.31 ± 0.51, respectively (p = 0.16). Also, there were no statistically significant differences of the body weight and BMI between the two groups.

**Table 1:** Subject characteristics at the baseline.

	Jinlida (n = 92)	Placebo (n = 94)
Diabetes duration (yr)	5.68±3.58	6.18±4.10
Age (yr-old)	55.18±9.13	55.81±9.93
Male (%)	53 (57.6)	54 (57.4)
Height (cm)	166.86±7.33	167.03±8.22
Weight (kg)	72.20±12.05	73.11±12.52
BMI	25.80±3.05	26.13±3.59
Waist circumference (cm)	89.17±9.78	89.52±10.38
Systolic BP (mmHg)	123.57±11.17	125.38±10.24
Diastolic BP (mmHg)	79.93±7.09	81.04±6.42
Heart rate (beat/min)	74.48±7.94	76.02±9.56
***Metformin use***		
Length of use (m)	33.53±29.20	34.77±28.92
Daily dose (g)	1.21±0.47	1.31±0.51
***Laboratory data***		
HbA1c (%)	8.10±0.89	8.33±1.22
FPG (mmol/L)	8.88±1.98	9.33±2.11
2-h PG (mmol/L)	15.58±4.01	16.46±3.91
Fasting insulin (Mu/L)	13.00±13.84	13.72±13.22
Triglyceride (mmol/L)	2.02±1.06	2.02±1.25

### Effects on HbA1c

**Fig 3:**
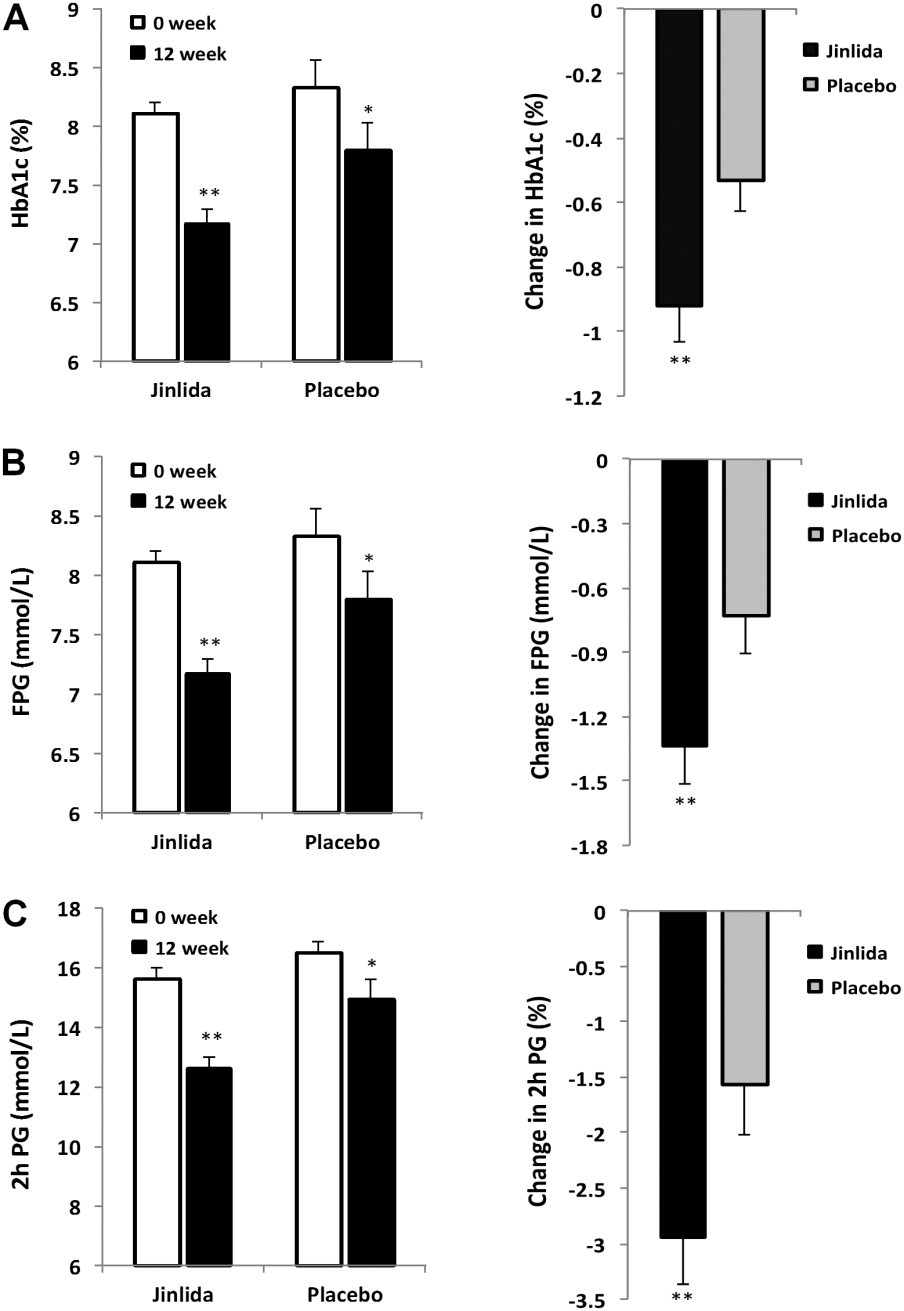
Changes in HbA1c, FPG and 2 h PG in the Jinlida group and placebo group. (A) Left: The mean HbAlc decreased significantly in both groups between the baseline and after treatment. Right: There was a very significant statistical difference in the change between the two groups at week 12. (B) Left: The mean FPG decreased significantly in both groups between the baseline and after treatment. Right: There was a very significant statistical difference in the change between the two groups at week 12. (C) Left: The mean 2 h PG decreased significantly in both groups between the baseline and after treatment. Right: There was a very significant statistical difference in the change between the two groups at week 12. Data presented as mean ± S.E. *p < 0.05. **p < 0.01.

At week 12, the HbA1c levels of the Jinlida group and placebo group were 7.18 ± 1.13 and 7.8 ± 1.44, respectively, both lower than those from week 0. Compared to the changes in the HbA1c from week 0, Jinlida group level was reduced by 0.92 ± 1.09 and placebo group level was reduced by 0.53 ± 0.94. The 95% CI was 0.69–1.14 for the Jinlida group vs. 0.34–0.72 for the placebo group. At week 12, there was a very significant HbA1c reduction between the Jinlida group and placebo group (p < 0.01) (Fig 3A). The outcome data obtained from the seven study centers (sites) generally was uniformly distributed across the various sites.

### Effects on FPG and 2h PG

Table 2 shows the levels of FPG at week 0, 4, 8 and 12, and the levels of 2h PG at week 0, 4, and 12, At week 12, both FG and 2h PG levels of the Jinlida group and placebo group were reduced from week 0. At week 12, there was a very significant FG reduction between Jinlida group and placebo group (p < 0.01; Fig 3B), and a very significant 2h FG reduction between Jinlida group and placebo group (p < 0.01; Fig 3C). The FG and 2h PG changes were consistent with the observed HbA1c data.

**Table 2:** Changes in FPG and 2h PG levels during the 12 week study.

	Jinlida (n = 92)	Placebo (n = 94)	*p* value
**FPG**			
Week 0	8.88±1.98	9.33±2.11	-
Week 4	8.24±1.99	9.11±2.85	-
Week 8	7.7±1.75	8.56±2.35	-
Week 12	7.54±1.61	8.6±2.27	-
Week 4—Week 0	-0.64±1.97	-0.21±2.14	0.161
Week 8—Week 0	-1.18±2.05	-0.76±1.97	0.160
Week 12—Week 0	-1.34±1.70	-0.73±1.70	0.006
**2h PG**			
Week 0	15.58±4.01	16.46±3.91	-
Week 4	14.69±4.28	15.84±4.94	-
Week 12	12.63±3.60	14.89±4.81	-
Week 4—Week 0	-0.89±4.27	-0.62±4.17	0.672
Week 12—Week 0	-2.95±3.99	-1.57±4.34	0.007

### Effects on HOMA-IR and HOMA-β

Homeostatic model assessment (HOMA) was performed.

**Fig 4:**
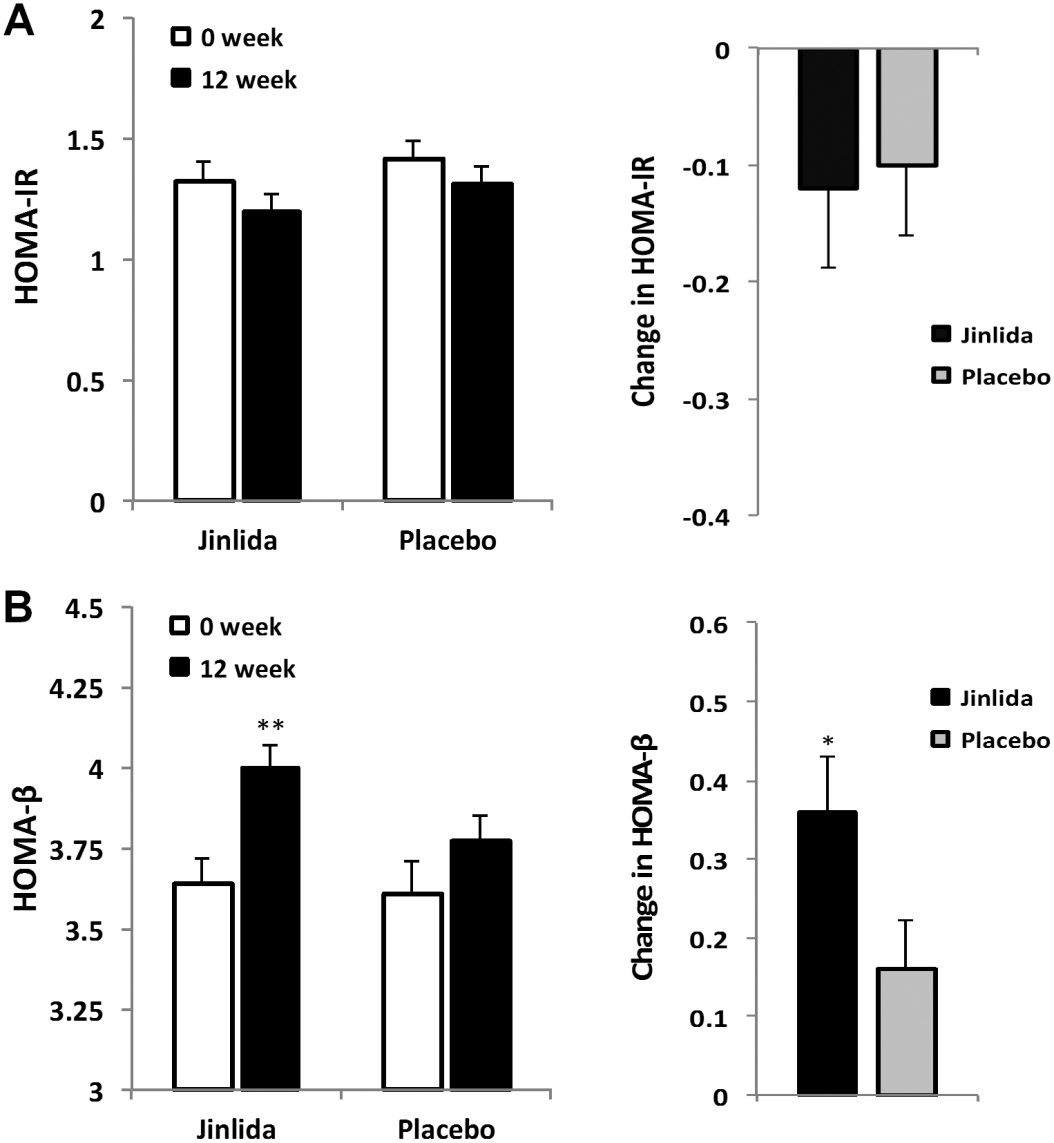
Changes in HOMA-IR and HOMA-β after 12 weeks treatment in the Jinlida group and placebo group. (A) Left: The mean HOMA-IR decreased somewhat in both groups between the baseline and after treatment. Right: Changes in HOMA-IR of the two groups. (B) Left: The mean HOMA-β increased very signigicantly in the Jinlida group between the baseline and after treatment. Right: There was a significant statistical difference in the change between the two groups. Data presented as mean ± S.E. * p < 0.05. **p < 0.01.

For insulin resistance, after 12 weeks of treatment, HOMA-IR was reduced in both the Jinlida group and placebo group. The HOMA-IR decreased by 0.12 ± 0.66 in the Jinlida group vs. by 0.01 ± 0.59 in the placebo group (p = 0.824; Fig 4A). The week 0, 4 and 12 data are presented in Table 3.

**Table 3:** Changes in HOMA-IR and HOMA-β values during the 12 week study.

	Jinlida (n = 92)	Placebo (n = 94)	*p* value
**HOMA-IR**			
Week 0	1.32±0.79	1.41±0.79	-
Week 4	1.27±0.69	1.41±0.71	-
Week 12	1.2±0.66	1.31±0.67	-
Week 4—Week 0	-0.05±0.62	0.01±0.48	0.567
Week 12—Week 0	-0.12±0.66	-0.1±0.59	0.824
**HOMA-β**			
Week 0	3.64±0.71	3.61±0.83	-
Week 4	3.85±0.68	3.74±0.84	-
Week 12	4.00±0.64	3.77±0.77	-
Week 4—Week 0	0.21±0.65	0.14±0.53	0.405
Week 12—Week 0	0.36±0.67	0.16±0.60	0.027

For β-cell function, after 12 weeks of treatment, HOMA-β increased in both the Jinlida group and placebo group. This increase was significantly higher in the Jinlida group (0.36 ± 0.67) compared to the placebo group (0.16 ± 0.6; p < 0.05) (Fig 4B). Week 0, 4 and 12 data are presented in Table 3.

### Effects on body weight, BMI, and waist circumference

**Fig 5:**
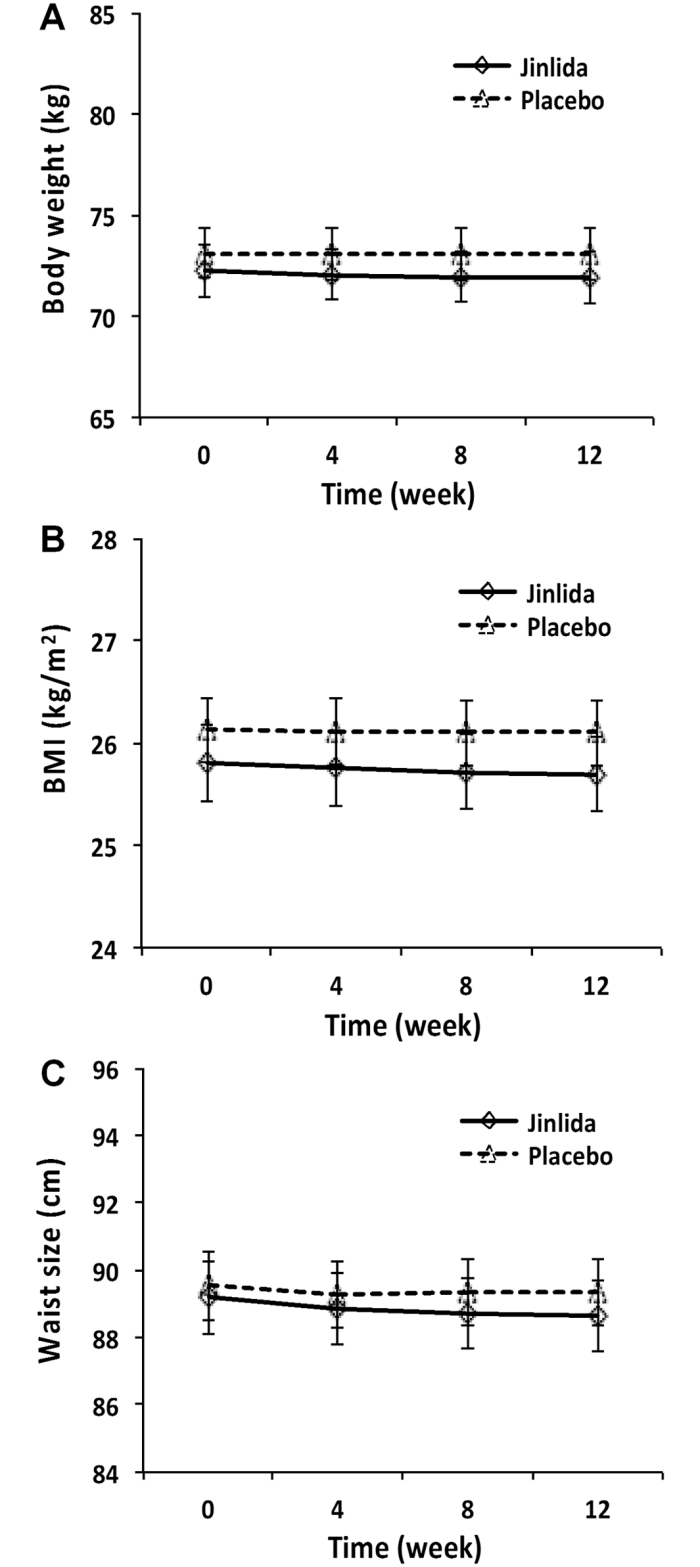
The mean values of body weight, BMI and waist circumference in the Jinlida group and placebo group. After 12 weeks of treatment, the body weight, BMI and waist size did not change significantly between the Jinlida and placebo groups and within the groups.

After 12 weeks of Jinlida treatment, compared to the placebo group, no statistical significance was observed in the body weight, BMI, and waist circumference (Fig 5). In addition, no obvious changes in food consumption or appetite were reported by the subjects.

### Safety analysis

No serious adverse events were reported in the Jinlida group and placebo group during the 12-week study period. No abnormal ECG, routine blood test, hepatic and renal function were recorded at week 12. In the placebo group, there were two cases of mild to moderate abdominal discomfort, which resolved without any medical attention.

## Discussion

Metformin is recommended as the first-line drug of choice in the treatment of type 2 diabetes. Endorsed by the American Diabetes Association and the Chinese Diabetes Society, metformin is a very commonly used medication clinically [5,6]. An epidemiological study with 97,315 Chinese type 2 diabetes patients showed that patients taking metformin monotherapy accounted for 30.8% of all the patients receiving oral antidiabetic medication in 2010 [20]. Metformin decreases hyperglycemia primarily by suppressing glucose production by the liver [21]. However, long-term use and/or dose escalating may lead to adverse effects, especially gastrointestinal irritation related symptoms [22]. The most serious adverse event of metformin use is lactic acidosis [23].

Currently, glycemic control in diabetic patients is far from satisfactory [2,7,8,24]. Often, as the disease progresses, additional oral hypoglycemic agent(s) or insulin need to be used. Based on the drug combination regime, metformin has been recommended for combination with different groups of oral antidiabetic medications for better glycemic control [5,6,25]. Various drugs have been previously used in combination with metformin, and variable positive results on lowering HbA1c have been published from many clinical trials [26–29]. These reports demonstrated that the dual oral therapy regimen is more effective than metformin alone for better glycemic control. More recently, Nauck et al. reported for patients received add-on dapagliflozin or glipizide for ver 18 weeks, in addition similar glycemic efficacy, dapagliflozin reduced weight and produced less hypoglycemia than glipizide in these type 2 diabetes patients inadequately controlled with metformin [30]. Fouqueray et al.’s 12-week trial used imeglimin, a new oral antidiabetic agent, as add-on therapy in the same diabetes patients population. Their data indicated that addition of imeglimin to metformin improved glycemic control and offered potential as a new treatment for type 2 diabetes [31]. In the above published combination metformin studies, the daily dose of metformin usually was 1.5–2.5 g. It is possible that cocktail therapy, including using herbal medications, would further increase the therapeutic effects in these patients.

Several previous clinical studies have been conducted in China using Jinlida for type 2 diabetes. Guo and Liu reported the effectiveness of Jinlida combined with metformin on type 2 diabetes patients. However that study was not a double-blind trial and only the patients with initial diabetes were enrolled [17]. Zhang et al. conducted a controlled study in type 2 diabetes patients in two groups: Jinlida plus placebo and metformin plus placebo. Significant differences have been observed in both groups in this 8-week trial [32]. The design of our present study is a 12-week multicenter controlled trial to evaluate if Jinlida enhances glycemic control of metforminin in type 2 diabetes patients, and we selected study patients whose diabetes was poorly managed with metformin alone.

In this study, our subjects engaged in standard diet control and exercise therapy. To meet the enrollment criteria, subjects should have been on steady dose metformin treatment for three months or longer, and their HbA1c should be ≥ 7.0%. For the enrolled subjects, the mean duration of metformin for the Jinlida group and placebo group were 34 and 35 months, respectively, and its mean daily dose was 1.2 and 1.3 g, respectively. The mean value of the HbA1c of the two groups was 8.1 and 8.3%, respectively. Our results showed that with Jinlida as an add-on therapy for 12 weeks compared to that of the placebo, the HbA1c was significantly reduced.

Further analysis of our data showed that the hypoglycemic effect of Jinlida as add-on therapy was more obvious in those subjects with a higher baseline HbA1c level. For those subjects with HbA1c 8.0–8.5% in the Jinlida and placebo groups, after 12 weeks, the HbA1c was reduced by 0.61% compared to the placebo group. Further, for those subjects with HbA1c > 8.5% in the Jinlida and placebo groups, after 12 weeks, the HbA1c was reduced by 1.35% compared to the placebo group.

In this trial, the changes in the FPG and 2h PG were consistent with the changes in the HbA1c in both the Jinlida and placebo groups. No significant body weight, BMI and waist circumference changes were reported from both groups. In addition, no serious adverse events with clinical significance were observed in our study subjects.

Our data showed that compared to the placebo group (metformin alone), Jinlida add-on therapy significantly improved β-cell function. In this study, the change in HOMA-IR did not reach statistical significance. Our further analysis suggested that for those subjects with insulin level > 20 μU/ml at baseline, the change in HOMA-IR appeared more obvious.

Traditional Chinese medicine has been used in the prevention and treatment of diabetes and diabetes related symptoms [11,33], and the herbal drug evaluated in this trial, Jinlida, contains over a dozen Chinese herbs. Our UPLC/MS chemical analysis data showed that puerarin is the major constituent in Jinlida. Previous studies have demonstrated that puerarin effectively reduced blood sugar levels, protected islet β-cell function, and helped to maintain metabolic homeostasis [34–36]. Other components from Jinlida, such as ginsenosides, have also shown potential antidiabetic activities [37,38]. In this study, we observed that Jinlida could significantly enhance the action of metformin in controlling the glycated hemoglobin level and increasing the β-cell function in our type 2 diabetes subjects.

Our study had some limitations, such as the 12-week study period was relatively short. Our data require further verification in interventional studies with a larger sample size, longer length of treatment and follow-up.

In summary, in this controlled trial, we showed the efficacy and safety of Jinlida in type 2 diabetes management. As a new candidate for add-on drug to metformin monotherapy, this Chinese herbal medicine may have a clinical utility in diabetes therapeutics.

## Supporting Information

S1 File Additional recorded information of this clinical trial. (PDF)

S2 File Study protocol. (PDF)

S1 CONSORT Checklist (PDF)
