# Supplementary material for: The Efficacy and Safety of Chinese Herbal Medicine Jinlida as Add-On Medication in Type 2 Diabetes Patients Ineffectively Managed by Metformin Monotherapy: A Double-Blind, Randomized, Placebo-Controlled, Multicenter Trial
Source: PLoS One. 2015 Jun 22;10(6):e0130550. doi: 10.1371/journal.pone.0130550 (PMC4476735; doi:10.1371/journal.pone.0130550)
Supplement: S1 File — (PDF) [file pone.0130550.s001.pdf]

Clinical research on Post-marketing Jinlida granule Assessment

Registration number：	ChiCTR-TRC-13003159
Date of releasing the registration number：	2013/04/20
Registration Status：	Prospective registration
Public title：	Clinical research on Post-marketing Jinlida granule Assessment
Scientific title：	The combined Jinlida granule and metformin treatment in patients with type 2 diabetes mellitus:A randomized, parallel-group, multicenter, double-blind and prospective study
Secondary ID：	

Applicant：	Lian Fengmei	Study leader：	Tong Xiaolin
Applicant telephone：	+86 010 88001402	Study leader's telephone：	+86 010 88001402
Applicant Fax：	+86 010 88001294	Study leader's fax：	+86 010 88001294
Applicant E-mail：	lfm565@sohu.com	Study leader's E-mail：	lfm565@sohu.com
Applicant website(voluntary supply)：	http://www.gamhospital.ac.cn/	Study leader's website(voluntary supply)：	
Applicant address：	No. 5, Bei Xian Ge, Xuan Wu District, Beijing	Study leader's address：	No. 5, Bei Xian Ge, Xuan Wu District, Beijing
Applicant postcode：	100053	Study leader's postcode：	
Applicant's institution：	Guang'anmen Hospital, China Academy of Chinese medical sciences		

Approved by ethic committee：	Yes		
Approved No. of ethic committee：	2013EC041	Approved file of Ethical Committee：	
Name of the ethic committee：	Guang'anmen Hospital of China Academy of traditional Chinese medicine ethics committee		
Date of approved by ethic committee：	2013/04/01		

Primary sponsor：	Guang'anmen Hospital, China Academy of Chinese medical sciences
Primary sponsor's address：	No. 5, Bei Xian Ge, Xuan Wu District, Beijing
Secondary sponsor：	No data added。
Source(s) of funding：	Shijiazhuang YiLing pharmaceutical co., LTD
Target disease：	Diabetes Mellitus
Target disease code：	
Study type：	Interventional
Study phase：	Post-market
Objectives of Study：	To evaluate the effects of Jinlida particles with Melformin on Glycated hemoglobin, blood sugar, insulin function, body mass index, waist circumference and the role of clinical symptoms in patients who are type 2 diabetes, Glycated hemoglobin as a main observing target, and to evaluate the safety of combined application, to provide clinical evidence for the combined treatment of traditional Chinese medicine and western medicine of diabetes.
Study design：	Randomized parallel control
Inclusion criteria	1.According to WHO diagnostic criteria of type 2 diabetes; 2. Into the group through the specification of diet control +exercise therapy + Stable doses of metformin for more than 3 months; 3.On an empty stomach blood sugar 7.0-13.9mmol/L, or after having meal two hours ≥11.1mmol/L; 4. Glycated hemoglobin≥7.0%; 5.18＜BMI＜40 kg/m2; 6.Ages ranged from 4 to 14 years old; 7.They signed the consent form.

Exclusion criteria：	1.Type 1 diabetes mellitus,Gestation diabetes mellitus and other special type ones; 2.In the past 3 months taking weight loss drugs, (including diet pills), oral any diabetes medication or insulin resistance in addition to the metformin outside; 3.With severe gastrointestinal disease, or be suffering from severe gastrointestinal diseases, such as: gastrointestinal ulcers, gastrointestinal bleeding, gastroparesis, pyloric stenosis, such as gastric bypass surgery; 4. Nearly one month have diabetes ketosis, ketosis acidosis ketosis high permeability diabetic coma, stress situation such as severe infection, operation; 5.With severe liver, renal insufficiency,Serum Creatinine (Cr)＞132.6umol/L (1.5mg/dL); 6.Patients with clinically uncontrolled hypertension. (Blood pressure ≥160/100mmHg); 7.TG＞5.65mmol/L; 8.Diabetic complication as the main symptom; 9.Psychopath, alcoholism and/or psychoactive substances, substance abuser and dependent; 10. pregnancy or lactation woman; 11.Allergic constitution.
Study execute time：	From2013-4-1To

Interventions：

Group：	treatment group	Sample size：	96
Intervention：	metformin +Jinlida granule 1bag, 3 times / day, warm boiling water	Intervention code：	
Group：	matched group	Sample size：	96
Intervention：	metformin	Intervention code：	

Countries of recruitment and research settings：

Country：	China	Province：	Jinlin	City：	Changchun City
Institution hospital：	Affiliated Hospital of Changchun University of Traditional Chinese Medicine	Level of the institution：	Tertiary		
Country：	China	Province：	Beijing	City：	Beijing
Institution hospital：	Xiyuan Hospital, China Academy of traditional Chinese Medicine	Level of the institution：	Tertiary		
Country：	China	Province：	Hebei	City：	Shijiazhuang
Institution hospital：	TCM Hospital of Shijiazhuang City	Level of the institution：	Tertiary		
Country：	China	Province：	Shanxi	City：	Taiyuan
Institution hospital：	Shanxi Provincial Hospital of Traditional Chinese Medicine	Level of the institution：	Tertiary		
Country：	China	Province：	Shanxi	City：	Taiyuan
Institution hospital：	Affiliated Hospital of Shanxi College of Traditional Chinese Medicine	Level of the institution：	Tertiary		
Country：	China	Province：	Hebei Province	City：	Shijiazhuang City
Institution hospital：	People's Liberation Army Bethune International Peace Hospital	Level of the institution：	Tertiary		

Outcomes：

Outcome：	glycosylated hemoglobin

Collecting sample(s) from participants：

Sample Name：	Blood	Tissue：	Vein
Fate of sample：	Destruction after use	Note：	

Recruiting status：	Pending	Participant age：	Min age 18 years; Max age 70 years
Randomization Procedure (please state who generates the random number sequence and by what method)：	Biometric unit provides a random number table	Gender：	Both
Blinding：			
Calculated Results ater the Study Completed：			
Organizer institution (leader institution)：			

Data collection Institution：

Data management Institution：

Data analysis Institution：

Return to list

Home | About ChiCTR | Trial Search | Document | Reg guide | Question

The world health organization international clinical trials registered organization registered platform

Copyright(c) (2005 - 2011) Chictr.org.All rights reserved. The Chinese clinical test registration center

Support: 543 social work center

蜀ICP备07505307号

Tips：it is recommended to use more than IE8.0 widescreen display resolution version using system.

站长统计
