# Supplementary material for: The Efficacy and Safety of Chinese Herbal Medicine Jinlida as Add-On Medication in Type 2 Diabetes Patients Ineffectively Managed by Metformin Monotherapy: A Double-Blind, Randomized, Placebo-Controlled, Multicenter Trial
Source: PLoS One. 2015 Jun 22;10(6):e0130550. doi: 10.1371/journal.pone.0130550 (PMC4476735; doi:10.1371/journal.pone.0130550)
Supplement: S2 File — (PDF) [file pone.0130550.s002.pdf]

CLINICAL STUDY ON POST-MARKETING RE-EVALUATION OF JINLIDA GRANULE

-----A RANDOMIZED DOUBLE-BLIND PLACEBO CONTROL MULTICENTRE CLINICAL RESEARCH ON COMBINATION OF JINLIDA GRANULE AND METFORMIN APPLIED TO TREATMENT OF TYPE 2 DIABETES MELLITUS

RESEARCH SCHEME

Scheme No.: yl-yxb10-lcsyfa-201301

Scheme Version No.: yl-yxb10-lcsyfa-201301-03

Chinese Cochrane Center Registration No.:

ChiCTR-TRC-13003159

Research Responsible Department: Guang'anmen Hospital, China Academy of Chinese Medical Science

Research leader: Professor Tong Xiaolin

Date: March 2013

Abstract of Research Scheme

Research topic	A randomized double-blind placebo control multicentre clinical research on combination of Jinlida Granule and Metformin applied to treatment of type 2 diabetes mellitus
Research objectives	Take glycated hemoglobin as main indicator to evaluate the effects of combination of Jinlida Granule and Melbiine on changes of glycated hemoglobin, blood sugar, insulin function, body mass index, waist circumference and clinical symptoms of type 2 diabetic patients, and discuss the safety of combined medication, in order to provide clinical basis for combined treatment of diabetes mellitus with traditional Chinese medicine and Western medicine.
Research design	Randomized, double-blind, placebo control, multicentre clinical research
Inclusion criteria and exclusion criteria	Inclusion criteria:<br>1. Meet WHO diagnostic criteria for type 2 diabetes mellitus.<br>2. Had taken standardized diet control + exercise therapy + stable administration of Metformin for more than 3 months before being enrolled;<br>3. Fasting glucose of 7.0-13.9mmol/L, or 2-hour post-meal blood glucose be or more than 11.1mmol/L;<br>4. Glycated hemoglobin not less than 7.0%;<br>5. 18 kg/m^2<BMI< 40 kg/m^2;<br>6. Aged between 18 and 70 years;<br>7. Had signed informed consent.<br>Exclusion criteria:<br>1. Type 1 diabetes mellitus, gestational diabetes mellitus and other special types of diabetes mellitus;<br>2. In the past 3 months, had taken medication to control body weight (including weight-loss drug), or oral administration of any anti-diabetic drugs or insulin except for Metformin;<br>3. Had a medical history or being suffered from severe gastrointestinal disease, such as, gastrointestinal ulcers, gastrointestinal bleeding, gastroparesis, pyloric stenosis, gastric bypass surgery, etc;<br>4. Had diabetic ketosis, ketoacidosis, nonketotic hyperosmolar diabetic coma, severe infection,

	surgery, or other stress response in recent a month;<br>5. Had serious hepatic and renal dysfunction, serum creatinine (Cr)> 132.6umol/L (1.5mg/dL);<br>6. Had uncontrolled hypertension (blood pressure be and more than 160/100mmHg);<br>7. Had blood fat (TG)> 5.65mmol/L;<br>8. Took diabetic complication as domination;<br>9. Had mental illness, alcohol addiction and/ or taken administration of psychoactive drug substance, drug abusers and addicts;<br>10. Pregnancy, lactation or being prepared pregnant women;<br>11. Had allergic constitution.	
Index of efficacy	Major index	Comparison of glycated hemoglobin between two groups at 12 weeks after treatment (Central Laboratory)
	Minor index	1. At 12 weeks after treatment<br>Insulin resistance index, insulin sensitivity index and β cell function index for two groups;<br>2. At 12 weeks after treatment<br>Fasting glucose and 2-hour post-meal blood glucose for two groups;<br>3. Clinical symptoms of patients in two groups at 12 weeks after treatment;<br>4. Body mass index (BMI);<br>5. Waistline.
Index of safety	Blood, routine urine test, hepatic and renal function, adverse event, physical examination	
Sample size	Total number of cases: 192 cases; trial group: 96 cases, control group: 96 cases.	
Dosage regimen	Trial group: Metformin + Jinlida Granule (1 bag per time, tid, drink with warm boiled water).<br>Control group: Metformin +placebo of Jinlida Granule (1 bag per time, tid, drink with warm boiled water).	
Course of treatment	12 weeks	
Statistical test	Biometric Room, Institute of Medicine, Hebei Medical University	
Expected progress	Initiated in March 2013; completed enrollment of subjects at the end of April, 2013; completed work summary in August, 2013.	

Research institution and Leader

	Name of hospital	Researcher
Lead institution	Guang'anmen Hospital, China Academy of Chinese Medical Science	Tong Xiaolin (main researcher)<br>Lian Fengmei (leader)
Collaborating institutions	Xiyuan Hospital, China Academy of Chinese Medical Science	Xia Chengdong
	Affiliated Hospital of Changchun University of Chinese Medicine	Piao Chunli
	Shijiazhuang Hospital of Traditional Chinese Medicine	Li Zhibin
	Shanxi Provincial Hospital of Traditional Chinese Medicine	Guo Junjie
	Bethune International Peace Hospital	Ma Licheng
	Affiliated Hospital of Shanxi College of Traditional Chinese Medicine	Zhao Lijuan

TABLE OF CONTENT(S)

1. BACKGROUND 6

2. OBJECTIVES 7

3. RESEARCH METHODS 7

3.1 RESEARCH DESIGN 7

3.2 SAMPLE SIZE 7

3.3 RANDOMIZED DESIGN 7

3.4 BLINDING LEVEL 7

3.5 EMERGENCY UNBLINDING 7

3.6 UNBLINDING PROVISION 7

4. RESEARCH OBJECTS 8

4.1 DIAGNOSTIC CRITERIA 8

4.2 POPULATION SELECTION 8

4.3 INCLUSION CRITERIA 8

4.4 EXCLUSION CRITERIA 9

5. INDEX OF EFFICACY 9

5.1 MAJOR INDEX 9

5.2 MINOR INDEX 9

5.3 INDEX OF SAFETY 9

5.4 SCREENING INDEX 9

6. CURATIVE CRITERIA 9

6.1 REFERENCE RANGE O GLYCATED HEMOGLOBIN IS DETERMINED ACCORDING TO TYPE 2 DIABETES PREVENTION GUIDE IN CHINA, 2010 EDITION 9

6.2 CHANGES OF INSULIN RESISTANCE INDEX, INSULIN SENSITIVITY INDEX, B CELL FUNCTION INDEX AND OTHER INDEXES 9

6.3 FASTING BLOOD GLUCOSE AND 2-HOUR POST-MEAL BLOOD GLUCOSE 10

6.4 CRITERIA ON CLINICAL CURATIVE EFFECT 10

6.5 BODY MASS INDEX AND WAISTLINE 10

7. CONDITION AND PROCEDURES OF SUBJECTS WITHDREW FROM RESEARCH 10

7.1 WITHDRAW DECIDED BY RESEARCHER 10

7.2 SUBJECT WITHDRAW RESEARCH VOLUNTARILY 10

7.3 SITUATION TO DISCONTINUE RESEARCH 10

8. INVESTIGATIONAL DRUGS 10

8.1 NAME, SOURCE, SPECIFICATION AND LICENSE NO. OF INVESTIGATIONAL DRUG 10

8.2 PACKAGING AND LABEL 11

8.3 DRUG INVENTORY AND RECOVERY 11

8.4 DRUG PRESERVATION 11

8.5 DRUG DISPENSATION AND RECOVERY 11

8.6 DRUG INVENTORY AND MANAGEMENT 11

9. THERAPEUTIC REGIMEN AND RESEARCH PROCESS 11

9.1 DOSAGE REGIMEN 11

9.2 RESEARCH PROCESS 11

9.3 PROVISION OF BASIC MEDICATION METFORMIN 12

9.4 COMBINATION THERAPY 13

10. OBSERVATION OF ADVERSE EVENT 13

10.1 DEFINITION13

10.2 CRITERIA FOR JUDGING INTENSITY OF ADVERSE EVENT 13

10.3 JUDGING STANDARD OF THE RELATION BETWEEN ADVERSE EVENTS AND INVESTIGATIONAL DRUG 13

10.4 JUDGMENT OF SERIOUS ADVERSE EVENT 13

10.5 FOLLOW-UP AND ADVERSE EVENT RECORD 14

10.6 ABNORMAL LABORATORY RESULTS 14

11. DATA MANAGEMENT 14

12. STATISTICAL ANALYSIS 14

12.1 ANALYSIS SET14

12.2 STATISTICAL ANALYSIS METHOD........ 15

13. QUALITY CONTROL 15

13.1 RESEARCH PROCESS MANAGEMENT 15

13.2 MEASURES TO IMPROVE CONSISTENCY OF OBSERVATION........ 15

13.3 MEASURES TO ENSURE SUBJECT COMPLIANCE 16

14. ETHICS-RELATED MATTERS 16

14.1 ETHICS REVIEW........ 16

14.2 INFORMED CONSENT FOR SUBJECTS........ 16

15. SCHEDULE OF RESEARCH 16

16. REFERENCES........ 16

17. APPENDIX 17

17.1 FLOW CHART OF RESEARCH 17

17.2 CLINICAL SYMPTOM SCORE 18

17.3 STANDARD OPERATING PROCEDURE FOR PROCESS IN CENTRAL LABORATORY........ 19

A RANDOMIZED DOUBLE-BLIND PLACEBO CONTROL MULTICENTRE CLINICAL RESEARCH ON COMBINATION OF JINLIDA GRANULE AND METFORMIN APPLIED TO TREATMENT OF TYPE 2 DIABETES MELLITUS

Research Text

1. Background

Today, diabetes mellitus poses an increasingly big threat to human health. Diabetic prevalence and the number of diabetic patients are growing rapidly. According to the International Diabetes Federation (IDF) statistics, there are 233 million diabetic patients worldwide, and with new-onset of 7 million patients each year. Based on the present rate of growth, it is estimated that there will be 380 million people suffering from diabetes worldwide in 2025. Currently, Asia is the region with the most diabetic patients, and the fastest growing rate is found in China, India and other developing countries.

In the early 1980s, diabetic prevalence rate in China was only 1%. The figure increased to 2.6% in 2002. China currently has at least 26 million diabetic patients, and there are similar number of candidates with hyperglycemia but not yet progressed to diabetes. IDF estimates that new-onset diabetes will reach 1.01 million in China each year. That is, there is 2,767 new-onset diabetic patients each day, or 115 new-onset diabetic patients an hour.

There are more diabetic patients, and diabetes can cause damage to vital organs of human body, which severely impact quality of life and limit patient's life, and greatly increase health care costs. Therefore, diabetes has posed a common threat to global human life and survival. Due to limited medical standards, diabetes cannot be cured recently. Therefore it should pay close attention to lifelong health of diabetic patients. The immediate goal of diabetic treatment is to control symptoms of diabetes, control blood sugar, and prevent acute metabolic complications; long-term goal is to achieve good metabolic control, prevent chronic complications, and improve quality of life of

diabetic patients.

Metformin is currently the preferred drug treatment of patients with type 2 diabetes. According to requirements of "Type 2 Diabetes Prevention Guide in China, 2010 Edition", Metformin should remain in treatment programs for patients without contraindications. However, during clinical treatment, when blood sugar is not within reference range, is to continue to increase the dose, or change to a multi-drug combination therapy, and when is the time to make a change, all of these are lack of evidence-based medical research evidence. Doctors often make judgments based on personal experience, resulting in poor treatment.

Data shows [1] that blood sugar begins to rise at 6 months to a year after Metformin monotherapy. A simple increase in single-dose can increase adverse reactions, but not improve efficacy. According to 2011 national survey on type 2 diabetes treated with oral medication, there was 10.7% patients taking Metformin monotherapy and 33.5% taking combination therapy with Metformin as basic drug in a total of 9872 patients being surveyed, but the overall compliance rate of glycated hemoglobin (<6.5%) was only 28%. The data indicates that it is insufficient to confirm Metformin as preferred therapeutic drug or basic drug in combination therapy for type 2 diabetes, meanwhile Metformin can easily cause hypoglycemia and weight gain with existing antidiabetic drugs such as sulfonylureas / glinides in combination drug treatment, which affects tolerability and compliance of patients, so that the effect is limited.

Traditional Chinese medicine can improve clinical symptoms associated with diabetes, improve quality of life, lower blood sugar and urine sugar, and reduce complications. So Chinese medicine collaterals theory has some advantages to guide treatment of diabetes and reduce its complications.

Collaterals theory suggests that the major pathogenesis of diabetes is unbalanced distribution and usage of body fluid and metabolic disturbance caused by splenic transfusion dysfunction, so it should make emphasis on nourishing Yin, benefiting qi, dissipating dampness, clearing heat, dredging collaterals and promoting splenic transfusion, and take “nourishing spleen and transfusing body fluid” as therapeutic guide. Jinlida Granule (approval No.: National Approval No. Z20050845) is the traditional

Chinese medicine with nourishing spleen, transfusing body fluid, benefiting qi and nourishing yin as the main rule of treatment. it is the innovation drug produced by Shijiazhuang Yiling Pharmaceutical Co., Ltd. It main indication is type 2 diabetes. Jinlida Granule achieved Hebei Province Science and Technology progress Award, and was included in the State's Key New Product Plans. It main components include ginseng, polygonatum sibiricum, rhizome atractylodis, sophora flavescens, radix ophiopogonis, radices rehmanniae, polygonum multiflorum, dogwood, poria, herba eupatorii, coptis chinensis, rhizome anemarrhenae, herba epimedii, radix salvia miltiorrhizae, radix puerariae, semen litchi, cortex lycii radicis and other Chinese herbal medicines. Basic research shows that the drug can reduce levels of blood sugar and serum triglyceride in the alloxan-induced diabetic rabbits, and decrease blood sugar in streptozotocin-induced diabetic rats. Single-center clinical study shows that Jinlida Granule can improve insulin sensitivity index for type 2 diabetic patients, and promote insulin resistance for type 2 diabetic patients.

Taking randomized double-blind, placebo control, multicentre clinical design, this research evaluates effect of combination of Jinlida Granule and Melbiine on improving glycosylated hemoglobin and blood sugar of type 2 diabetes mellitus, and explores its curative efficacy and clinical safety.

2. Objectives

Take glycosylated hemoglobin as main indicator to evaluate effect of combination of Jinlida Granule and Melbiine on changes of glycosylated hemoglobin, blood sugar, insulin function, body mass index, waist circumference and clinical symptoms of type 2 diabetic patients, and discuss safety of combination medication, in order to provide clinical basis for combined treatment of traditional Chinese medicine and Western medicine of diabetes mellitus.

3 Research Methods

3.1 Research design

Randomized, double-blind, placebo control, multicentre clinical research

3.2 Sample size

According to literature, the difference is 0.5% in reducing glycosylated hemoglobin for

combination of Metformin and Jinlida Granule comparing with single administration of Metformin, and standard deviation is 1.1%, as calculating with a ratio of 1:1 between two groups, taking α=0.05 and β=0.20, and sample size of 77 cases in each group (a total of 154 case). The total number of cases is 192 with consideration of expulsion rate of 20%.

Note: sample size is calculated with PASS2008 software.

3.3 Randomized design

This study takes randomized grouping design. Subjects are grouped at a ratio of 1:1 for trial group and control group, which can maximize the advantages of double-blind trial, and eliminate bias produced by unbalanced allocation ratio. This kind of allocation can enhance curative balance for each group, and control other factors that may affect the test results.

The biometric experts who are independent of the data management and statistical analysis use SAS statistics software package on the computer to produce the codes. By employing the stratified randomization method, the codes for the trial group and the control group are generated at random according to a 1:1 ratio. The length of the selected block and the seed parameter of random numbers initial value shall be sealed in the blind codes as confidential data. On the basis of this random number, the statistician who is independent of the trial will encode the drugs. The clinical trial centers will use the drugs according to the assigned drug code and the patient’s order in the group. The blind codes are in duplicate and shall be kept in the national clinical research base for drug where the sponsor and the major investigator come from.

3.4 Blinding level

This research adopts double-blind design that researchers, patients and supervisors are unknown of the drug subjects being taken, and this method can greatly eliminate bias produced during clinical research and explanation of clinical results.

3.5 Emergency unblinding

In double blind study, each blind code is set up with an emergency letter, the content of which includes the group this numbered subject belongs to and situation of medical treatment. In the meanwhile of the drug encoding, the biometric experts who are in charge of generating of the blind codes will prepare an emergency letter for each drug

code. The group which the drug code actually belongs to is sealed in the letter. The emergency envelopes are sent to the centers along with the corresponding drugs. The chief investigator in that center is responsible for keeping the envelope. When a serious adverse event occurs, there is an urgent need for unblinding. At this time, the chief investigator in that center shall open the emergency envelope corresponding to its drug code. Once the envelope is opened, the case is considered as drop-out case. However, if there are any adverse reactions, this case shall be recorded in the adverse reaction analysis. The double-blind research will be invalid when the blind codes is leaked or when the opening and reading rate of the emergency envelopes exceeds 20% in the process of clinical research.

3.6 Unblinding provision

In this study, subjects of trial group and control group are at a ratio of 1:1, and two-unblinding method is adopted. Data files will be locked after blind review and being confirmed with reliability and accuracy, and then the first unblinding will be carried out. The first unblinding only states the group that each case belongs to (such as group A or group B), but does not indicate which is the trial group or control group. Results of the first unblinding should be entered computer by statistical professionals, and statistically analyzed after connecting with the data file. The second unblinidng will be carried out after statistical analysis to clear the treatment scheme received by each group.

4. Research Objects

4.1 Diagnostic criteria

Type 2 diabetic disease is diagnosed according to standards established by WHO Committee of Diabetic Experts in 1999.

(1) Symptoms of diabetes (typical symptoms include polydipsia, polyuria and unexplained weight loss) and 1) random blood glucose (level of blood glucose at any time without consideration of the last meal) be and more than 11.1mmol/L (200mg/dl), or 2) fasting blood glucose (fasting state refers to the state without adding calories for at least 8 hours) be and more than 7.0mmol/L (126mg/dl), or 3) at 2 hours after glucose load, blood glucose be and more than 11.1mmol/L (200mg/dl).

(2) The subjects, who are without diabetic symptoms, should detect blood glucose at another day to make a definite diagnosis.

4.2 Population selection

(1) The patient, who takes administration of Metformin at a stable dosage for more than 3 months, and underwent strict diet and exercise control, is with blood glucose and gycosylated hemoglobin out of reference range.

Appendix:

① Diet control method: take balanced and reasonable dietary guidance, calculate required total calories according to height, weight and activity level of patient, calculate ideal calories with total intake calorie and actual consumed calorie, balance dietary by nutritionist, and take low-fat diet. Then nutritionist develops recipes and varieties of food, which should be carried out by patients. Calories provided by common fat do not exceed 30% of total calories of diet, acceptable daily intake of saturated fat should not exceed 10% of total calories, and do not eat fried foods or whole milk foods; calorie provided by carbohydrates should take 55% to 60% of total calories, mainly are complex carbohydrates, which should be equally distributed in three meals; calories provided by protein takes 15% to 20% of total calories, mainly is high-quality protein. Quit smoking and limit alcohol, the salt intake should be less than 6 g for hypertensive patients. Take 1600 calorie as example, distribution ratio of three major nutrients is that protein takes 15% to provide 240 calorie, and equivalents to 60 g protein; carbohydrate takes 60% to provide 960 calories, and equivalents to 240 g carbohydrate; fat takes 25% to provide 400 calories, and equivalents to 100 g fat. According to *Chinese Food Composition Table* or *Food Exchange List*, clinical nutrition is calculated, and exchanged to specific amount of daily food. Calorie distribution ratio of three meals is 1/5, 2/5 and 2/5 for breakfast, lunch and dinner of diabetic patients, respectively.

Total required calories for adult diabetic patients of different body figures each day (kJ/kg)

Body figure	Heavy physical labor	Moderate physical labor	Light physical labor	Rest
Thin (below	188 to 209	147 to 167	105 to 146	83.6 to 104.5

normal weight 20% or more)				
Normal weight	147 to 167	126 to 146	84 to 126	62.7 to 83.6
Obesity (excessive weight 20% or more)	126 to 146	105 to 126	104.5 to 83.6	62.7 to 83.6

②Exercise therapy: sport events are mainly aerobic exercises characterized by rhythmic movement of large muscle group, as walking, gymnastics, jogging, tennis, cycling, and down the stairs, badminton, swimming, aerobics, etc. It needs 3 to 6 motor units each day according to following sports exchange table. Moderate exercise intensity is recommended that post-exercise heart rate +170-age (years), and at least 3 to 5 days every week.

Exercise intensity	Sports event	Required exercise time for consuming a unit calorie
Light	Walk, ride standing, shopping, cleaning the room, weeding	30min
Moderate intensity	Walk, bath, go down stairs, ride a bike, laundry, dance	20min
High intensity	Jogging, climb stairs, old disco, volleyball, table tennis	10min
Extremely high intensity	Climbing, play football, swimming, play basketball, rope skipping	5min

(A motor unit is equivalent to consume 80 kcal calories)

4.3 Inclusion criteria

(1). Meet WHO diagnostic criteria for type 2 diabetes mellitus.

(2). Had taken standardized diet control + exercise therapy + stable administration of Metformin for more than 3 months before being enrolled;

(3). Fasting glucose of 7.0-13.9mmol/L, or 2-hour post-meal blood glucose be or more than 11.1mmol/L;

(4). Glycated hemoglobin be or more than 7.0%;

(5). 18 kg/m^2<BMI< 40 kg/m^2;

(6). With age of 18 to 70 years old;

(7). Had signed informed consent.

4.4 Exclusion criteria

(1). Type 1 diabetes mellitus, gestational diabetes mellitus and other special types of diabetes mellitus;

(2). In the past 3 months, had taken medication to control body weight (including weight-loss drug), or oral administration of any anti-diabetic drugs or insulin except for Metformin;

(3). Had a medical history or being suffered from severe gastrointestinal disease, such as, gastrointestinal ulcers, gastrointestinal bleeding, gastroparesis, pyloric stenosis, gastric bypass surgery, etc;

(4). Had diabetic ketosis, ketoacidosis, nonketotic hyperosmolar diabetic coma, severe infection, surgery, or other stress response in recent a month;

(5). Had serious hepatic and renal dysfunction, serum creatinine (Cr)> 132.6umol/L (1.5mg/dL);

(6). Had uncontrolled hypertension (blood pressure be and more than 160/100mmHg);

(7). Had blood fat (TG)> 5.65mmol/L;

(8). Took diabetic complication as domination;

(9). Had mental illness, alcohol addiction and/ or taken administration of psychoactive drug substance, drug abusers and addicts;

(10). Pregnancy, lactation or being prepared pregnant women;

(11). Had allergic constitution.

5. Index of Efficacy

5.1 Major index

Glycated hemoglobin (being detected in Central Laboratory)

5.2 Minor index

(1) Insulin resistance index, insulin sensitivity index and β cell function index for two groups at 12 weeks after treatment;

(2) Fasting glucose and 2-hour post-meal blood glucose for two groups at 12 weeks after treatment;

(3) Clinical symptoms of patients in two groups at 12 weeks after treatment;

(4) Body mass index (BMI);

(5) Waistline.

5.3 Index of safety

Blood, routine urine test, hepatic and renal function (alanine aminotransferase (ALT), aspartate aminotransferase (AST), blood urine (BUN) and serum creatinine (Cr)), frequency of hypoglycemia and other adverse events.

5.4 Screening index

Blood fat (triglyceride, TG)

6. Curative Criteria

6.1 Reference range o glycated hemoglobin is determined according to *Type 2 Diabetes Prevention Guide in China, 2010 Edition*.

Difference of glycated hemoglobin between two groups at 12 weeks after medication

6.2 Changes of insulin resistance index, insulin sensitivity index, β cell function index and other indexes

HOMA model formula is adopted to evaluate β cell insulin secretory function:

1) β cell function index (HOMA-IS)= 20×FINS/(FPG-3.5);

2) Insulin resistance index (HOME-IR)=(FINS×FPG) /22.5;

3) Insulin sensitivity index (IAI) is calculated with Li Guangwei formula: IAI=1/(FINS×FPG).

6.3 Fasting blood glucose and 2-hour post-meal blood glucose

Changes of fasting blood glucose and 2-hour post-meal blood glucose are compared between two groups by corresponding statistical method at 12 weeks after medication.

6.4 Criteria on clinical curative effect

Disappear: clinical symptom disappears before treatment, and score is 0.

Improve: clinical symptom improves before treatment, and score decrease, but not 0.

Invalid: clinical symptom without improvement or even becoming worse, and score increase.

6.5 Body mass index and waistline

Changes of body mass index and waistline are compared between two groups by corresponding statistical method at 12 weeks after medication.

7. Condition and Procedures of Subjects Withdrew from Research

7.1 Withdraw decided by researcher

Subject withdrew the study indicates that the enrolled subject cannot continue research at certain condition, and researcher decides the subject to withdraw from research process.

(1) During research, subject has severe acute and chronic complications of diabetes (diabetic ketoacidosis, hyperosmolar nonketotic syndrome, lactic acidosis, hypoglycemic coma, acute myocardial infarction, acute stroke, etc) or special physiological changes (positive HCG) that he or she is inadequate to participate in research continuously.

(2) During research, subject is with poor compliance, dosage of medication is less than 80% or more than 120% of specified amount.

(3) During research, subject violates program rules, and takes oral administration of other hypoglycemic drugs without approval of researcher.

(4) After drug administration of 4 weeks, the subject, who is with measured value of fasting blood glucose higher than 13.9 mmol/L at consecutive 2 times within a week, should withdraw from study.

7.2 Subject withdraw research voluntarily

According to informed consent, subject has the right to withdraw research; if the subject does not put forward the idea of withdrawing research clearly, but losses to follow up for refusal taking medication or detection, he or she also withdraws researcher (or “off”). The reason of withdraw should be comprehended and recorded. Such as feeling poor about curative effect; intolerable adverse effects; discontinue participating in clinical research for different reasons; economic factors; or loss to follow up without obvious reasons, etc.

The history record table should be preserved for the subject who withdraws research for any reasons, the last detection result can be considered as final result, and curative efficacy and adverse effect should take full data set analysis.

7.3 Situation to discontinue research

Research suspension refers to the clinical research discontinues during process, not ends as planned. The purpose of research suspension is to protect interests of subjects, ensure quality of research and avoid unnecessary economic losses.

(1) If there is serious security problem, the research must discontinue immediately.

(2) If the drug is with poor curative efficacy, or even ineffective, or without clinical significance, the research should discontinue, to avoid delay for effective treatment of subjects or unnecessary economic loss.

(3) If the clinical research program has a major mistake, and is difficult to evaluate drug effects; or a better designed program has significant deviation during implementation, and cannot continue to carry out to evaluate drug efficacy, the research should discontinue.

8. Investigational Drugs

8.1 Name, source, specification and license No. of investigational drug

1. Name of investigational drug: Jinlida Granule

Source: Shijiazhuang Yiling Pharmaceutical Co., Ltd

Medical components: ginseng, polygonatum sibiricum, rhizome atractylodis, sophora flavescens, radix ophiopogonis, radices rehmanniae, polygonum multiflorum, dogwood, poria, herba eupatorii, coptis chinensis, rhizome anemarrhenae, herba epimedii, radix salvia miltiorrhizae, radix puerariae, semen litchi and cortex lycii radicis

Property: its content is brown to black brown granule, and tastes bitter.

Specification: 9 g per bag

License No.: ******

2. Name of investigational drug: Placebo of Jinlida Granule

Source: Shijiazhuang Yiling Pharmaceutical Co., Ltd

Medical components: malt, lactose, dextrin

Property: its content is brown to black brown granule, and tastes bitter.

Specification: 9 g per bag

License No.: ******

8.2 Packaging and label

Jinlida Granule is a kind of granule preparation; its placebo has the same appearance. Outer packaging is as follows, and each small box has 9 bags.

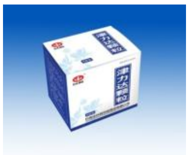

8.3 Drug inventory and recovery

At each visit, observe doctor should record receive amount, dosage and return amount of drug in detail, to determine the compliance of patient, which should be recorded in case report table.

8.4 Drug preservation

Investigational drug should be locked and kept in safe and controllable indoor areas, and keep dry. Each center should specify a medical management person to keep and manage drugs.

8.5 Drug dispensation and recovery

After passing screening, subjects should be given drugs by researcher in order. At each dispensation, researcher should immediately and accurately fill in drug dispensation record, record amount of dispensed drugs, and remind subjects taking remain drugs back at the next visit; the researcher should recover and check the amount of recovery drugs, which should be recorded in drug dispensation/ recovery record. After research, all remained drugs should be returned to sponsor, and recorded in recovery table of investigational drug.

8.6 Drug inventory and management

At each follow-up, observe doctor should record faithfully and in detail whether the patient takes treatment on time at designated place, to determine the degree of subject compliance. The record should be made at format medical record. Medication compliance=total amount of actual drug use/ total amount of required drug use by program ×100%. At completion, research drug administrator is responsible for giving back remaining drugs to biding unit or destroying remaining drugs. Research drugs should be preserved in locked specific cabinet at room temperature.

9. Therapeutic Regimen and Research Process

9.1 Dosage regimen

Trial group: Metformin + Jinlida Granule (1 bag per time, tid, drink with warm boiled water).

Control group: Metformin +placebo of Jinlida Granule (1 bag per time, tid, drink with warm boiled water).

Variety, usage and dosage of Metformin are same as original.

Oral administration of research drugs is recommended to take after the meal. If the patient had intolerable adverse events, which may be caused by research drugs determined by researcher, the patient should discontinue research drug treatment.

9.2 Research process

Visit 1: Enrollment period (the third day before being enrolled to the day just before enrollment)

If the patient meets all eligible criteria of this scheme, he or she should complete all examinations. The patient, who meets inclusion criteria and exclusion criteria, should be randomized grouped.

The following contents should be completed at visit 1:

(1) Subject signs informed consent;

(2) Obtain medical history;

(3) Obtain demographic data;

(4) Take complete physical examination, acquire information of vital signs, and fill in baseline information of disease;

(5) Fasting blood-glucose, 2-hour post-meal blood glucose, glycated hemoglobin, fasting insulin detection;

(6) Blood sample for detection of glycated hemoglobin should be preserved in refrigerator at -20℃ in Central Laboratory;

(7) Laboratory safety detection (blood routine test, routine urine test) and ECG;

(8) Blood fat (total cholesterol) detection;

(9) Review inclusion/ exclusion criteria;

(10) Record concomitant drugs;

(11) Dispense drug, and complete drug dispensation record.

Visit 2: Randomized treatment stage (at the end of the fourth week):

(1) Inquire symptoms to determine subject continue or discontinue study;

(2) Fasting blood-glucose, 2-hour post-meal blood glucose, glycated hemoglobin, fasting insulin detection;

(3) Blood sample for detection of glycated hemoglobin should be preserved in

refrigerator at -20℃ in Central Laboratory;

(3) Laboratory safety detection (blood routine test, routine urine test) and ECG;

(4) Record clinical symptoms table;

(5) Inquire and record concomitant medication and adverse events;

(6) Dispense drug, and complete drug dispensation record.

Visit 3: Randomized treatment stage (at the end of the eighth week):

(1) Inquire symptoms to determine subject continue or discontinue study;

(2) Fasting blood-glucose;

(3) Record clinical symptoms table;

(4) Inquire and record concomitant medication and adverse events;

(5) Dispense drug, and complete drug dispensation record.

Visit 4: At the end of research (at the end of the 12th week)

(1) Fasting blood-glucose, 2-hour post-meal blood glucose, glycated hemoglobin, fasting insulin detection;

(2) Blood sample for detection of glycated hemoglobin should be preserved in refrigerator at -20℃ in Central Laboratory;

(3) Laboratory safety detection (blood routine test, routine urine test) and ECG;

(4) Record clinical symptoms table;

(5) Inquire and record concomitant medication and adverse events;

(6) Fill in drug dispensation and recovery record, and make an inventory of recovery drug;

(7) Fill in research completion record.

Visit 5: (required follow-up and visit)

The patient, who had persist adverse events, laboratory abnormity with clinical significance, abnormal vital signs, etc, should take necessary follow-up and obtain detection results at follow-up visit. Researcher can take follow-up or telephone visit according to specific situation.

9.3 Provision of basic medication Metformin

At research period, Metformin should take original variety and dosage, and cannot change; if there is hyperglycemia, the subject should withdraw research. If there is

hypoglycemia, the subject should take lower dosage of Metformin. Adverse events should be recorded.

9.4 Combination therapy

(1) The patient should take reasonable diet and exercise for diabetic patients to change lifestyle. Besides of research drugs, the patient should not take other hypoglycemic drugs, insulin or Chinese and Western medicines for treating diabetes, or the drug greatly influencing blood glucose (as corticosteroids, thyroxine, tricyclic antidepressants, weight-reducing drugs etc.).

(2) The drugs, which have administrated to treat other concomitant diseases, should not change the variety and dosage in principle.

(3) Detailed record should be made for taking other drugs to treat other concomitant disease during research.

10. Observation of Adverse Event

10.1 Definition

(1) Adverse event (AE): any unfavorable medical incident happens since the time when the subject signs the informed consent and enrolls the trial to the finish of the last follow-up is judged as adverse event, no matter whether the medical incident is associated with the investigational drug or not.

(2) Significant adverse event: besides severe adverse event, any adverse event happens and causes the use of pointed medical measures (e.g. drug withdrawal, reducing dosage and expectant treatment) and hematological and/or other laboratorial anomaly.

10.2 Criteria for Judging Intensity of Adverse Event

All clinical adverse events occurred in this clinical research will be recorded on the adverse events page of CRF and classified according to the intensity. In order to have a unified standard, the intensity grade of events is as follows:

Slight perceptible malaise but not affect daily activities

Moderate stronger malaise leading to affect or reduce daily activities

Severe malaise that unable to work or carry out daily activities

It needs to pay attention to distinguish the severity and intensity of adverse events. Severity is used to describe intensity and it may not be a serious adverse event (SAE).

For example, the expression of headache is possibly severe in intensity, but it can't be regarded as SAE unless it meets the criterion of SAE.

10.3 Judging standard of the relation between adverse events and investigational drug

The causality analysis of the relation between all adverse events and the investigational drug is judged according to five grades, i.e. certainly related, probably related, possibly related, possibly unrelated and definitely unrelated. The first three grades are judged drug adverse reaction. In the causality analysis we consider the following five aspects.

1）Do the time of beginning to use the medicine and the time of appearance of Adverse Drug Reaction (ADR) have reasonable precedence relation (appearance during using medicine)?

2）Does the suspected ADR match the already-known ADR of the drug (conformity with the literature)?

3）Can the suspected ADR be explained by influences of combining with drug, original drug, clinical state of patients or the effect of other therapies (other explanations)?

4）Do the suspicious ADR disappear or reduce after drug withdrawal or decrement (withdrawal symptom)?

5）Does suspicious ADR appear again after taking the same medicine (taking again, appearing again)?

The investigator should evaluate the possible existing connection among adverse events, investigational drugs and the combining drugs. Please refer to the table below.

Factors	Appearance during using medicine	Conformity with the Literature	Other explanations	Withdrawal symptom	Taking again, appearing again
Certainly Related	＋	＋	－	＋	＋
Probably Related	＋	＋	－	＋	?
Possibly Related	＋	＋	±	±	?

Possibly Unrelated	+	–	±	±	?
Definitely Unrelated	–	–	+	–	–

10.4 Judgment of serious adverse event

(1) Definition of serious adverse event

Serious adverse event refers to any clinical event that cue obvious damage, contraindication, side effects or need more cares. Any adverse event which meets one or more of the following criterion will be identified as serious adverse event.

- Death.
- Life risk (refers to the patient in this event is in danger of death when the event occurs, but it doesn't include the events that would probably cause the patient to die if there is progressive aggravation).
- Causing admission or longer stay at hospital.
- Causing permanent or obvious labor capacity loss or disability.
- Congenital anomaly or birth defect.

(2) Clinical events which haven't cause death, life risk or admission, but after proper medical judgment, it is believed that the events can cause the potential harm to the patient or it is necessary to give the patient drug treatment or surgery so as to avoid causing the above states should be also identified as serious adverse event.

10.5 Follow-up and adverse event record

Adverse events, especially the events which have a connection with the investigational drugs, should be followed up until they return to the baseline or tend to be steady. If follow-up can't make the adverse events back to the baseline or to be steady, then it should be recorded and explained in the CRF. Any serious adverse events occurred in the clinical trial must be reported to the sponsor and major research institution within 24 hours. At the same time, the investigator must fill in the SAR report and record the occurrence time, seriousness, duration, measurement and turnover.

10.6 Abnormal laboratory results

The investigator should judge whether the abnormal laboratory results are of clinical significance and provide possible interpretation. The reported adverse event which causes the abnormal laboratory results should also be recorded as adverse events in ADR. Abnormal laboratory results which include at least one of the following items are of clinical significance, and they should be recorded on the adverse events page of CRF as independent diagnosis (excluding the abnormal laboratory result caused by the adverse events which has already been reported):

- Combining with clinical symptom
- Leading to the change of investigational drug
- Requiring to change the combination therapy and (or) other therapeutic measure.

11. Data Management

Researchers should load original observation record of subject into case report table correctly, completely, clearly and timely. Supervisor should confirm that the trail is carried out according to trail scheme, and all case report tables are filled correctly and completely, the data is consistent with original materials. If there is any mistake or omission, researcher should correct it immediately. After being checked, the case report table should be submitted to data administrator of clinical trial by supervisor.

Data administrator should establish a database before getting the first case report table. Each case report table should be checked preliminarily by data administrator, and then entered to database by two operators independently. These two inputting results are compared by corresponding software. If there is any inconsistency, please find out reason and correct it. Data administrator should conduct data check according to range of each index and mutual relations, including range check, logical check, etc. all mistake content and modified results should be recorded in detail and properly kept. If it is necessary, all or sampling index (especially for key index) in database should be manually checked, and verified with case report table.

If there is any problem, data administrator should inform supervisor immediately, and ask researcher to make an explanation. Query table should be used to record various

questions and answers, and kept properly for reference.

After completion of above works, main researchers, sponsor unit, trail statistical worker and data administrator should conduct blind review, to confirm analysis set each case belonging to, treatment of missing value, determination of outlier, etc. After the blind review and confirming that a correct data base has been established, the data should be locked. The first unblinding is conducted, and blind answer and database should hand over to statistical professionals to conduct statistical analysis.

12. Statistical Analysis

12.1 Analysis set

Full analysis set (FAS): it refers to the data set of all subjects participating in randomization and being evaluated after medication at least once. For those subjects who fail to observe all healing effect evaluation, we conduct the data transfer according to LOCF (last observation carry forward) principle.

Per protocol set (PPS): it refers to the data set of all subjects meeting inclusion criteria, inconsistent with exclusion criteria and completing therapeutic scheme. Analysis (per protocol, PP) is taken on data of all cases consistent with research scheme, with good dependency and completion of filling in contents specified by CRF.

Safety set (SS): It refers to the data set of subjects who are grouped at random, take the research drug and at least have one safety assessment after the baseline.

Baseline comparative analysis and curative effect analysis are conducted based on FAS and PPS, and safety evaluation is conducted according to SS.

12.2 Statistical analysis method

Statistical analysis is made by SPSS19.0 software. All statistical tests should be performed by two-sided test. P value less than or equal to 0.05 are considered as statistical difference.

Descriptive analysis: enumeration data is represented as constituent ratio; measurement data is expressed as mean, standard deviation, median, minimum value and maximum value.

Statistical analysis: appropriate method should be taken according to type of index. Comparison of quantitative data among groups should be performed by *t* test or *Wilcoxon* rank sum test; covariance analysis should be performed for consideration of central effect and concomitant variable; categorical data is calculated with chi-square test or Fisher's

exact test; grade data is calculated by *Wilcoxon* rank sum test or CMH test.

Detailed statistical method is recorded in statistical analysis plan.

13. Quality Control

13.1 Research process management

(1) Research drugs are preserved and managed by research institution, and all drugs should be kept in hospital pharmacy. The person designated as "research drug administrator" is responsible for drug recipient, counter custody, recovery, return and destruction of research drugs for dropout and losing follow-up patients. *Clinical Research Drug Usage Record* should be established. *Clinical Research Drug Usage Record* should include: dispensation date of research drug, subject name, research drug code, severity level, amount of given drugs, signature of researcher, amount of remaining drugs, return to sponsor and other items.

(2) After Informed Consent being signed by subjects, clinical researcher should get research drugs from "research drug administrator", and dispenses to subject according to visit order. Relevant information should be recorded in *Clinical Research Drug Usage Record*.

(3) Provision of detecting glycated hemoglobin in Central Laboratory: if the glycated hemoglobin is 7.0% or more detected in branch center, the patient can be grouped with consideration of other indexes. The designated person is responsible for taking venous blood; anticoagulant vacuum blood vessels are used to collect double blood samples at 3 ml, which should be kept in low temperature freezer at -20℃. After completion of tasks in hospital, supervisor should contact cold chain company, and one sample is sent to Central Laboratory. The detection results by Central Laboratory are as the criterion for statistical analysis, and the other sample is as backup copy.

(4) Provision of standard meal: to reduce difference of 2-hour post-meal blood glucose detection, research group should provide instant noodles at the same specification and brand to each research center, and oil bag should be discarded

(5) Anticoagulant blood vessel is used to collect blood samples for detection of glycated hemoglobin in central laboratories of different hospitals.

13.2 Measures to improve consistency of observation

(1) Participants to observe and collect clinical data should be with higher professional knowledge and skills, and relatively permanent.

(2) With training before research, researchers completely comprehend clinical research scheme and plans. The description of subjective symptoms should be objective, without induction or indication. The regulated objective index should be examined by designated method at regulated time of scheme. Adverse events and unforeseen side effects should be observed and followed up. Case Record Table should be filled in, there is without null term or arbitrary alteration.

(3) Significant deviation and data out of acceptable range should be verified, and investigator should make necessary instructions.

(4) Each test item should be marked with unit of measurement.

(5) Each clinical research unit should designate person to take regular examination of clinical research progress, and carefully verify the information and records.

(6) If necessary, bidding unit should organize clinical mid-meeting, to check preliminary work, analyze problems in clinical research, and propose rectification.

(7) Determination of clinical diagnosis and curative evaluation after treatment should be made by a doctor in charge and a resident doctor at least. Observation Table should be filled in by resident doctor or the one with above title.

13.3 Measures to ensure subject compliance

(1) Do a good work for subject that he or she can comprehend significance of research and take medication under supervision and urgent of family members;

(2) The patient, who is with poor efficacy and cannot take medication on time, should be followed up by researcher commonly.

(3) Auditor should appoint supervisor, who should ensure the benefit of subjects, accuracy and completion of research record and report data, and the research follows the approved clinical scheme, drug quality management practice and regulations.

14. Ethics-related Matters

14.1 Ethics review

This clinical study must follow Helsinki Declaration and related clinical research laws and regulations in China. Before research, Ethics Committee of research responsible hospital should review research scheme, and implement research scheme after signing approval documents. Suggestions of Ethics Committee should be: agree, which refers to

consent after taking necessary correction; disagree, which refers to terminate or suspend approved research.

14.2 Informed consent for subjects

Before being enrolled in the research, each patient or the designated representatives should be introduced about objectives, characteristics, procedures and possible benefits and risks of the research by doctor completely and comprehensively. The patient has right to withdraw research at any time. Each patient is given a written informed consent (as an appendix in scheme) before research, and agrees its content after understanding. After signing Informed Consent, the patient can be enrolled in clinical study. As original clinical research data, the Informed Consent should be preserved for reference.

15. Schedule of Research

The first ten days of March, 2013 Hold Scheme Preliminary Meeting participated by research units

The middle ten days of March, 2013 Research drug and prepare materials

The last ten days of March, 2013 Pass ethical review in Guang'anmen Hospital, initiate Branch Center.

The first ten days of April 2013 Screen grouping cases

The last ten days of April, 2013 Complete randomized grouping of all cases

July 2013 The centers complete the follow-up of all cases

August 2013 Complete data entry and blind review

August 2013 Complete the summary report of the trial

16. References

[1] PREVENTION PROGRAM RESEARCH GROUPN. Engl J Med 2002;346:393-403.

[2] World Health Organization. Definition, Diagnosis and Classification of Diabetes Mellitus and its Complications. In: Report of a WHO Consultation. WHO/NCD/NCS/99.2. Geneva:WHO,1999.

17. Appendix

17.1 Flow chart of research

Phase / Item	Grouping	Treatment stage			Follow-up
Times of visit	**1**	**2**	**3**	**4**	**5**
Visit time	**-3 to 0 day**	**4 weeks**	**8 weeks**	**12 weeks**	
Baseline information					
Demographic data	√				
Common clinical data	√				
Inclusion criteria and exclusion criteria	√				
Informed consent	√				
Medical history and treatment history	√				
Drug combination	√	√	√	√	
Curative index					
Glycated hemoglobin	√	√		√	
Fasting	√	√	√	√	

blood-glucose					
2-hour post-meal blood glucose	√	√		√	
Fasting insulin	√	√		√	
Table of clinical symptoms	√	√	√	√	
Body mass index and waistline	√	√		√	
Exclusion criteria					
Blood fat (triglyceride)	√				
Security index					
Blood routine test and routine urine test	√	√		√	
Hepatic and renal function	√	√		√	
Electrocardiogram	√	√		√	
Record of adverse event		√	√	√	
Random treatment					
Random grouping	√				

Dispense drugs	√	√	√		
Quantitative statistics of drugs		√	√	√	
Summary of research results				√	

Follow-up *: At the end of treatment, due to adverse events or laboratory persistent, abnormal vital signs and abnormity of other vital signs, researchers considered that it was necessary to take follow-up visits in order to obtain the measurement results. Researchers can take follow-up visits by telephone based on the specific situation.

17.2 Clinical symptom score

It is enacted according to *Clinical Guiding Principle of New Traditional Chinese Medicine* issued by Ministry of Health in 1993 and *Clinical Guiding Principle of New Traditional Chinese Medicine for Treatment of Diabetes* in *Clinical Guiding Principle of New Traditional Chinese Medicine* published by China Medical Science Press in 2002.

1) Thirsty and polydipsia

Without symptom	0
Slight increase of water intake compared with before	2 points
Over half times increase of water intake compared with before	4 points
Over one time increase of water intake compared with before	6 points

2) Lassitude and asthenia

Without symptom	0
Easy to feel tired, but can carry on physical labor	2 points
Mental fatigue, reluctantly take daily labor	4 points
Feel weakness of limbs, and cannot take daily labor	6 points

3) Polyphagia and easy to hungry

Without symptom	0

Obvious sense of hunger	1 point
Intolerable hunger before the meal	2 points
Intolerable hunger and often concomitant with hypoglycemia reaction	3 points

4) Polyuria:

Without symptom	0
Slight increase of urine volume compared with usual, and daily urine volume within 2 L	1 point
Increase of urine volume, and daily urine volume of 2 to 3 L	2 points
Obvious increase of urine volume, and daily urine volume more than 3 L	3 points

5) Dry mouth and throat

Without symptom	0
Slightly dry mouth and throat, which can be alleviated after drinking a little water	1 point
Dry mouth and throat, which can be alleviated after drinking much water	2 points
Unbearable dry mouth and throat, which is difficult to alleviate after drinking water	3 points

6) Spontaneous sweating

Without symptom	0
Slight sweating after exercise, and clothes at slightly damp condition	1 point
Skin is slightly damp even without exercise, and sweating become worse after low intensity exercise.	2 points
Always sweating, and can find water stain after exercise	3 points

7) Feverish palms and planter centre

Without symptom	0
Feverish palms and planter centre at night, occasionally feel upset	1 point
Hot palms and planter centre, sometimes feel	2 points

upset	
Burning hot palms and planter centre, want to hold cold materials, and feel upset and restless all days	3 points

8) Constipation:

Without symptom	0
Stool becomes hard, once a day	1 point
Hard bound stool, once two to three days	2 points
Dry stool and difficult to discharge, about more than four days once	3 points

Make a detail record of tongue and pulse situations, but not give a score.

17.3 Standard operating procedure for process in Central Laboratory

Standard Operating Procedure for Preparation, Detection and Cold Chain Transportation of Blood Sample

1. Objective

Standardize preservation, transportation, received condition and process of blood sample.

2. Scope of application

A randomized double-blind placebo control multicentre clinical research on combination of Jinlida Granule and Metformin applied to treatment of type 2 diabetes mellitus

Glycated hemoglobin is detected in Central Laboratory, which is responsible for preparation, detection and transportation of blood sample.

3. Simple flow

**Collect blood sample**	**Responsible party: researcher in branch center**
**Preservation**	
**Turn over**	
**Responsible party of transportation**	**Express company**
**Receive**	**Responsible party: Guang'anmen Hospital**
**Chemical analyst**	

4. Preparation and preservation

Each center should prepare blood sample within 2 hours after collection according to standard operating procedure for preparation and preservation of blood sample. Blood sample should be kept in freeze pipe. Two pages of self-adhesive label with drug No. should be attached to freeze pipe, and the other is attached in reserved sample record (Table 1), which should be fill as requirement.

Preparation of blood sample:

Draw 3 ml venous blood
EDTA anticoagulation vessel
Make patient label
Be kept in refrigerator at -20℃
All samples should be inspected within 2 weeks after being collected.

Notes:

1) Make a detection under the condition without inflammation or infection (metabolic stability), to reduce individual differences;

2) Patient preparation: venous blood is collected at sitting position from Am 8:00 to 9:00 after remaining fasting for more than 12 hours.

3) Preparation of blood sampling: take two anticoagulant blood vessels to collect 6 ml venous blood, to avoid hemolysis and lipemia (please make a note, if there is).

Each vessel should contain more than 3 ml blood, one for detection in central laboratory and the other as reserved sample. Both vessels should be marked with same No. as that in record table (Table 1), including name, sex, age, sample collecting time and collect unit.

4) Transportation and preservation of sample: whole blood frozen sample vessel should be kept in refrigerator at -20℃, and the longest preservation period is 6 months. The sample should be kept in ice box during transportation. Refrigerator temperature should be recorded for reference each day.

5. Transportation

After researchers are enrolled, supervisor is responsible for calling Shanghai Shengsheng Logistics Company at 15800437256; reporting pickup account and specific

pickup address, contact person, telephone, address for service and so on.

Please check and prepare samples on the day appointed by express company, and express worker should pack up and transfer samples after getting to hospital.

Researchers should fill in **Blood Sample Receipt**, which should be attached with **Copy of Sample Record** for express delivery, (original record should be returned to researchers by supervisor during the next inspection) for check and use. Data of sample, Sample Record and Transference Table should be identical.

Express Company should sent samples to Central Laboratory within 24 hours.

6. Receive

On the day of service, the blood samples should be checked, numbered and put in storage by staff in Central Laboratory, who should immediately fill in Sample Transference Record, and open the package for examination and record the conditions of samples at the time of receiving samples.

7. Detection

Central Laboratory should complete detection within 10 working days after receiving samples, and submits the detection results to the sponsor and supervisors. Within a week after receiving the detection results, the supervisor should provide feedback results to corresponding research center.
